# Physical exertion at work and addictive behaviors: tobacco, cannabis, alcohol, sugar and fat consumption: longitudinal analyses in the CONSTANCES cohort

**DOI:** 10.1038/s41598-021-04475-2

**Published:** 2022-01-13

**Authors:** Nadine Hamieh, Alexis Descatha, Marie Zins, Marcel Goldberg, Sébastien Czernichow, Nicolas Hoertel, Marie Plessz, Yves Roquelaure, Frédéric Limosin, Cédric Lemogne, Joane Matta, Guillaume Airagnes

**Affiliations:** 1 INSERM, Population-Based Epidemiological Cohorts Unit, UMS 011, 16 Avenue Paul Vaillant Couturier, 94807 Villejuif, France; 2 Poison Control Center, Academic Hospital CHU Angers, F-49000 Angers, France; 3 Univ Angers, Centre Hospitalier Universitaire CHU Angers, Université de Rennes, INSERM, École des hautes études en santé publique, Institut de recherche en santé, environnement et travail Irset UMR_S 1085, F-49000 Angers, France; 4 Université de Paris, Faculty of Health, School of Medicine, Paris, France; 5 Université de Paris, AP-HP, Hôpital européen Georges-Pompidou, Service de Nutrition, Paris, France; 6 Université de Paris, AP-HP, Hôpital Corentin-Celton, DMU Psychiatrie et Addictologie, Service de psychiatrie et d’addictologie de l’adulte et du sujet âgé, Issy-les-Moulineaux, INSERM, Institut de Psychiatrie et Neurosciences de Paris (IPNP), UMR_S1266, Paris, France; 7 INRAE, Centre Maurice Halbwachs (ENS, EHESS, CNRS) UMR 8097, Paris, France; 8 University of Angers, Centre Hospitalier Universitaire d’Angers, Université de Rennes, Centre de consultations de pathologie professionnelle et santé au travail, F-49000 Angers, France; 9 Université de Paris, AP-HP, Hôpital Hôtel-Dieu, DMU Psychiatrie et Addictologie, Service de Psychiatrie de l’adulte, INSERM, Institut de Psychiatrie et Neurosciences de Paris (IPNP), UMR_S1266, Paris, France; 10 Université de Paris, AP-HP.Centre-Université de Paris, DMU Psychiatrie et Addictologie, Paris, France

**Keywords:** Health occupations, Risk factors

## Abstract

We examined the prospective association of physical exertion at work with subsequent tobacco, cannabis, alcohol use, and sugar and fat consumption. Volunteers of the French population-based CONSTANCES cohort currently employed were included from 2012 to 2017 for tobacco and cannabis outcomes (n = 100,612), and from 2012 to 2016 for alcohol and sugar and fat outcomes (n = 75,414). High level of physical exertion at work was defined as a score ≥ 12 at the Rating Perceived Exertion Borg scale. Substance use was self-reported and diet rich in sugar and fat was obtained from principal component analysis and analyzed as quartiles. Generalized linear models computed odds of substance use and sugar and fat consumption at follow-up according to baseline physical exertion at work, while adjusting for sociodemographic factors, depressive symptoms and baseline level of consumption. High physical exertion was associated with tobacco use with dose-dependent relationships. It was also associated with increased odds of cannabis use at least once per month compared to no use in the past and with increased odds of diet rich in sugar and fat. Hence, the role of physical exertion at work on tobacco and cannabis use and diet rich in sugar and fat should be tackled for information and prevention strategies.

## Introduction

Substance use are the first preventable cause of premature death worldwide^1^. If left untreated, they could lead to somatic disorders (e.g., cancers and cardiovascular disorders)^2,3^, psychiatric disorders (e.g., mood disorders and suicide)^4–7^ and social deprivation including occupational issues (e.g., absenteeism, work accident and job loss)^8,9^. At the population level, the most concerned substances are tobacco, cannabis and alcohol use. Among former substance users, prevention of relapse is also a major issue^10^. Sugar and fat overconsumption are also highly prevalent in western countries and they share common vulnerability factors with substance use^9^.

Substance use and sugar and fat consumption could be driven by environmental factors such as occupational factors^11^. For instance, substance use and craving for sugar and fat increase after losing a job^12^ or retiring^13^. However, work stress may increase the likelihood of substance use and relapse in former users^14^. For instance, high job demand has been associated with increased risks of using substances in the workplace^15^. In this study, we focused on physical exertion at work and its associations with tobacco, cannabis and alcohol use and high sugar and fat consumption. Such working conditions have already been associated with detrimental physical health consequences (e.g. musculoskeletal disorders)^16^. However, its potential consequences on substance use and sugar and fat consumption have not been examined yet, to the best of our knowledge. Since occupational health strategies exist to deal with physical exertion at work, their benefits could be extended to decreasing the burden of such detrimental behaviors.

We took advantage of the CONSTANCES French national population-based cohort to examine prospectively the relations between physical exertion at work and tobacco, cannabis, alcohol use and sugar and fat consumption in a large sample of employees randomly recruited from various social and occupational backgrounds^17^. In addition, sociodemographic factors and depressive symptoms were also reported, allowing to take into account their potential confounding and/or moderating roles. We hypothesized that physical exertion at work is associated with increased substance use and sugar and fat consumption. Since gender and age are related to different patterns of substance use, sugar and fat consumption and occupational factors^18^, we also planned to examine changes in this association according to sex and age groups.

## Methods

### Study design

The French population-based CONSTANCES cohort enrolled volunteers since 2012, aged 18–69 years at baseline, and according to a random sampling scheme stratified on age, gender, socioeconomic status and region of France^17^. Among the different procedures conducted with participants, they completed annual self-administered questionnaires on their lifestyle, health, social, and personal characteristics. All the procedures are detailed at www.constances.fr. CONSTANCES has obtained the authorization of the National Data Protection Authority, was approved by the Institutional Review Board of the National Institute for Medical Research (Authorization number 910486) and was performed in accordance with relevant guidelines and regulations. All participants signed an informed consent form to be included in the cohort.

### Study populations

**Figure 1:**
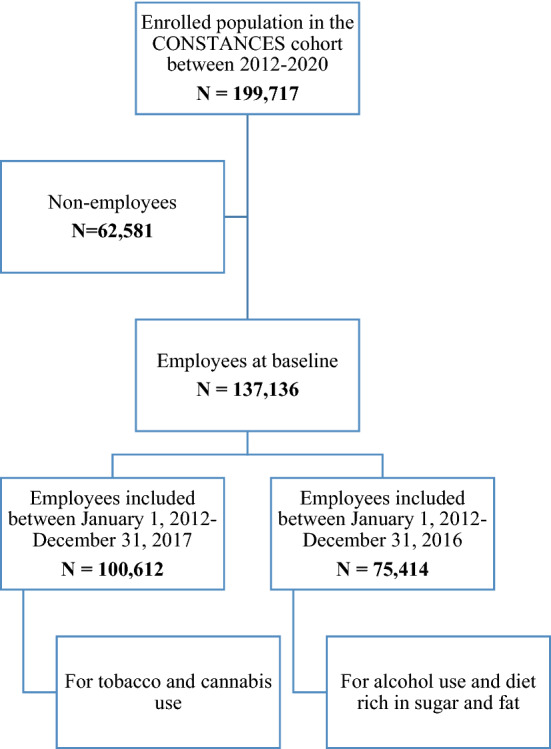
Cohort flow chart in the CONSTANCES cohort.

A total of 199,717 volunteers were enrolled in the CONSTANCES cohort between January 6, 2012 and January 8, 2020. Among these volunteers, those who were not employed at baseline (n = 62,581) were excluded by selecting those who reported to have currently a job. Since outcomes were available at different periods of follow-ups, individuals included after January 1, 2018 (n = 36,524) were excluded when studying the tobacco and cannabis outcomes, to allow for 1-year of follow-up duration (since the last follow-up date of these outcomes was in 2018 at the time the present study was conducted). Regarding alcohol and sugar and fat outcomes, volunteers included after January 2017 (n = 61,722) were excluded since the last available follow-up endpoint was in 2017 for these outcomes. Data on sugar and was available only at baseline and five years later in 2017. Hence, a total of 100,612 participants were included for studying tobacco and cannabis use and 75,414 for studying alcohol use and diet rich in sugar and fat (Fig. 1, Supplementary Table S1).

### Assessment of physical exertion

Physical exertion was assessed using the Rating Perceived Exertion (RPE) Borg scale at baseline. The RPE Borg scale is a scale ranging from 6 to 20 with 6 ‘No effort at all’ and 20 ‘Exhausting’ that assesses the perceived physical exertion by asking the volunteers to rate their intensity of physical effort during a typical working day^19^. The distribution of the RPE Borg scale by year of enrollment is shown in Supplementary Fig. S1 and Supplementary Table S2. Volunteers with a score of ≥ 12 were considered to be exposed to high physical exertion according to the ‘French National Research and Safety Institute for the Prevention of Occupational Accidents and Disease’^20^.

### Assessment of tobacco use

Smoking status (i.e., Never smokers; Former smokers; Current smokers) was self-reported, as well as daily tobacco consumption in number of cigarettes per day among current smokers. Thus, we further categorized the current smokers into three categories: Current light smokers (< 10 cigarettes/day); Current moderate smokers (10–18 cigarettes/day) and Current heavy smokers (> 19 cigarettes/day)^18^ and computed a variable with 5 modalities as followed: Non-smokers; Ex-smokers; Current light smokers; Current moderate smokers and Current heavy smokers.

Since we also planned to examine the risk of relapse, the following variables were also computed:

- ‘Relapse of tobacco use among ex-smokers at baseline’ with ‘No’: remained non-smokers at follow-up and ‘Yes’: became current smokers at follow-up irrespective of the tobacco use intensity.
- ‘Changing status among current smokers at baseline’ with ‘Ex-smokers’: stopped smoking at follow-up; ‘Current light smokers’: remained current light smokers at follow-up; ‘Current moderate smokers’: remained current moderate smokers at follow-up and ‘Current heavy smokers’: remained current heavy smokers at follow-up.
- ‘Changing status among ever smokers (i.e., former smokers and current smokers) at baseline with: ‘Smokers at baseline and follow-up’: remained current smokers at follow-up irrespective of the consumption; ‘Smokers at baseline and stopped at follow-up’: became ex-smokers at follow-up; ex-smokers at baseline and follow-up and “Ex-smokers at baseline and started smoking at follow-up’.
- Changes in number of cigarettes per day among continuous current smokers, computed as the difference between number of cigarettes per day at follow-up and at baseline.

### Assessment of cannabis use

From three questions assessing the frequency of cannabis use at follow-up among no cannabis users for at least 12 months at baseline, the following categorical variable was computed: no use in the past 12 months at both assessments (reference category), cannabis use less than once a month at follow-up, cannabis use at least once per month at follow-up.

### Assessment of alcohol use

Alcohol use was assessed continuously and categorically. It was assessed continuously as the difference between the weekly consumption at follow-up and at baseline. The weekly consumption was computed in drinks per week at baseline and at follow-up based on a reporting of all the alcoholic beverages consumed the previous week by multiplying the daily number of drinks per day by 7. Then, alcohol use was assessed categorically according to the World Health Organization risk level classification (WHO, 2000): “Low risk” (1–27 drinks/week in men and 1–13 in women); “No use” and “At risk” (≥ 28 drinks/week in men and ≥ 14 in women).

### Assessment of diet rich in sugar and fat

Diet rich in sugar and fat was assessed using the principal component analysis (PCA) of a 32-item qualitative food frequency questionnaire. These items represented the daily frequency of the consumed food (i.e., sugar, meat, cheese, yogurt and others) on a scale from 0 to 4 with 0 being ‘rarely or never’ and 4 ‘5 or more’. From these items, three factors were generated: diet rich in sugar and fat, traditional diet and diet rich in low fat protein (Supplementary Table S3). Diet rich in sugar and fat which was our variable of interest, was assessed as quartiles variables using the indices obtained by the principal component analysis while considering the first quartile as the reference group.

### Assessment of covariables at baseline

Sociodemographic factors included age, sex, occupational grade (low: manual and clerical; medium: technical; high: managerial positions), educational level and household income. Educational level and household income were assessed using self-reported questions on the highest obtained diploma based on the International Standard Classification of Education 2011^21^, and on total household net monthly income, respectively. In addition, socioeconomic status was measured as a continuous variable by the PCA of educational level, occupational grade and household income for the supplementary analyses. From these items, one factor was generated which was socioeconomic status (Supplementary Table S4).

Depressive symptoms were assessed using the French version of the CES-D scale which has a high internal consistency (α = 0.90 in the CONSTANCES cohort) and volunteers with a score of ≥ 19 were considered to be clinically depressed according to the validated threshold for the French version (sensitivity and specificity > 85% for the diagnosis of major depression)^22^.

### Statistical analysis

Generalized linear regressions were computed to study the associations between physical exertion at work (exposure) and tobacco, cannabis, alcohol use and diet rich in sugar and fat (outcomes). All the analyses were carried by using unadjusted and fully-adjusted models. The fully-adjusted models were adjusted for all the covariables mentioned above baseline level of consumption for the substance chosen as the outcome. However, regarding the changes in tobacco and cannabis use between follow-up and baseline, there was no adjustment for baseline level of consumption since the construction of the outcome variable already included this information. Since educational level, occupational grade and household income could be correlated, we tested for the correlations and multicollinearity between these three variables in the fully-adjusted models. No threat for multicollinearity was found (Supplementary Tables S5, S6); neither a difference in the interpretation of the analyses when occupation was removed from the models (Supplementary Table S7). Hence, all these three variables were included in the fully-adjusted models. Cochran–Mantel–Haenszel tests were performed to search for trends between physical exertion and the outcomes.

Then, the analyses were stratified for sex and age, separately. The following age groups were considered: 18–29; 30–39; 40–49; 50–59; ≥ 60.

Sensitivity analyses were performed for diet rich in sugar and fat since this outcome had only one available follow-up endpoint, 2017 and was assessed over 5 years. First, we additionally adjusted for duration of follow-up in the model. Duration of follow-up was assessed continuously as the difference between 2017 and at baseline. Second, we tested for statistical interaction between physical exertion at work and duration of follow-up since duration of follow-up could be an effect modifier between the association between physical exertion and diet rich in sugar and fat. If a significant interaction was found, stratified analyses were done.

As supplementary analyses, Cox proportional hazard regression were used to calculate the hazard ratios (HR) and 95% confidence intervals (CI) of physical exertion at work and the aforementioned outcomes in the unadjusted and fully-adjusted models. Each participant contributed person-time from the start date of our study, January 6, 2012, until death, the occurrence of the substance use or diet rich in sugar and fat or the last follow-up questionnaire (2017 or 2018 depending on the outcome), whichever occurred first.

Since job type/quality could be associated with the aforementioned outcomes, we tested for statistical interactions between occupational grade, type of job contract and type of work and physical exertion. We further examined the association between physical exertion and outcomes in stratified analyses. Type of job contract and type of work were assessed as binary measures: temporary versus permanent contract and part time versus full-time, respectively.

Lastly, although each of educational level, household income and occupational grade has different relationships with health, and it is well known that there is a significant decoupling between education and income as well no collinearity between these three covariates was found in this study, we run additional analyses by replacing them by socioeconomic status.

Missing data were handled by multiple imputations. All *P*-values were two-sided with an α = 0.05. All statistical analyses were undertaken using the SAS system software (version 9.4, SAS Institute, Cary, NC).

## Results

The baseline characteristics of the 100,612 and 75,414 employees included between 2012 and 2017 (to study tobacco and cannabis use) and 2012–2016 (to study alcohol use and diet rich in sugar and fat), are respectively summarized in Table 1. The prevalence of high physical exertion at work was 33.3% among employees included between 2012 and 2017 (n = 33,579) and 32.8% among those included between 2012 and 2016 (n = 24,795). All the covariables were associated with physical exertion at work. For instance, employees with higher physical exertion were older, more likely to be men, had a lower occupational grade, a lower education and a lower income and less likely to report depressive symptoms (all *P* < 0.0001).

**Table 1:** Baseline characteristics of 100,612 and 75,414 employees included between 2012 and 2017 (to study tobacco and cannabis use) and 2012–2016 (to study alcohol use and diet rich in sugar and fat), respectively by exposure to physical exertion in the CONSTANCES cohort study.

	Total	Between 2012 and 2017		*P*	Total	Between 2012 and 2016		*P*
		High physical exertion	No high physical exertion			High physical exertion	No high physical exertion	
	N = 100,612	N = 33,579	N = 67,033		N = 75,414	N = 24,795	N = 50,619	
Mean (SD) age, years	43.6 (10.9)	43.4 (11.2)	43.6 (10.8)	** < 0.0001**	43.9 (10.9)	43.7 (11.2)	43.9 (10.8)	** < 0.0001**
**Age, %**				** < 0.0001**				** < 0.0001**
18–29	13.0	38.0	62.0		12.6	37.6	62.4	
30–39	24.7	30.6	69.4		23.8	30.0	70.0	
40–49	29.9	33.0	67.0		29.9	32.7	67.3	
50–59	26.5	34.9	65.1		27.6	34.1	65.9	
≥ 60	5.9	30.0	70.0		6.1	29.7	70.3	
**Male sex, %**	47.0	34.5	65.5	** < 0.0001**	47.3	33.8	66.2	** < 0.0001**
**Occupational grade, %**				** < 0.0001**				** < 0.0001**
Low	38.1	50.7	49.3		37.5	50.5	49.5	
Medium	29.1	36.4	63.6		29.3	35.6	64.4	
High	32.8	10.6	89.4		33.2	10.6	89.4	
**Educational level, %**				** < 0.0001**				** < 0.0001**
< Baccalaureate	35.9	53.2	46.8		36.5	52.0	48.0	
≥ Baccalaureate	64.1	22.3	77.7		63.5	21.9	78.1	
**Household income, %**				** < 0.0001**				** < 0.0001**
< 2100 €/month	19.9	50.8	49.2		19.9	50.5	49.5	
≥ 2100 €/month	80.1	29.0	71.0		80.1	28.5	71.5	
Depressive symptoms*, %	15.6	43.1	56.9	** < 0.0001**	15.6	43.0	57.0	** < 0.0001**

Independent t-tests and Chi-square tests were computed for continuous and categorical variables, respectively.

Significance values are given in bold.

*Depressive symptoms were defined as having a CES-D score ≥ 19.

### Association between physical exertion at work, substance use and diet rich in sugar and fat

#### Tobacco use

In ex-smokers at baseline, high physical exertion increased the odd of relapsing (aOR 1.13, 95% Confidence Interval (CI) 1.02–1.24) (Table 2). This association vanished in women when stratified by sex (Supplementary Table S8).

**Table 2:** Association between high physical exertion at work and tobacco use at 1-year of follow-up among employees in the CONSTANCES cohort study, 2012–2018 (odds ratios (ORs), and 95% confidence intervals, CI).

	N (%)	Unadjusted model	Fully-adjusted model*
		OR (95% CI)	OR (95% CI)
**Tobacco use**			
Relapse of tobacco use among ex-smokers at baseline	30,916		
No	25,218 (81.6)	1.00	1.00
Yes	5698 (18.4)	**1.37 (1.29–1.45)**	**1.13 (1.02–1.24)**
Changing status among current smokers at baseline	20,078		
Ex-smoker	5787 (28.8)	1.00	1.00
Current light smoker	8406 (41.9)	**1.54 (1.43–1.66)**	**1.21 (1.12–1.31)**
Current moderate Smoker	4751 (23.7)	**2.14 (1.97–2.32)**	**1.34 (1.23–1.47)**
Current heavy smoker	1134 (5.6)	**2.47 (2.17–2.81)**	**1.54 (1.33–1.78)**
*P-*trend	** < 0.0001**		
Changing status among ever-smokers at baseline	50,994		
Smoker at baseline and remained smoker at follow-up	14,291 (28.0)	1.00	1.00
Smoker at baseline and stopped at follow-up	5787 (11.3)	**0.56 (0.52–0.60)**	**0.78 (0.73–0.84)**
Ex-smoker at baseline and stopped at follow-up	25,218 (49.5)	**0.60 (0.58–0.63)**	**0.85 (0.81–0.90)**
Ex-smoker at baseline and started smoking at follow-up	5698 (11.2)	**0.83 (0.78–0.88)**	0.94 (0.87–1.01)
*P-*trend	** < 0.0001**		

		*ß* (95% CI)	*ß* (95% CI)
Number of cigarettes/day among current smokers at baseline	20,078	− 0.02 (− 0.18; 0.14)	**0.35 (0.20; 0.51)**

Categories of current smokers were defined as: light smokers (< 10 cigarettes/day), moderate smokers (10–18 cigarettes/day) and heavy smokers (> 19 cigarettes/day).

Relapse was defined as: no (remained non-smokers at follow-up) and yes (became current smokers at follow-up).

Changing status among current smokers was defined as ex-smokers (stopped smoking at follow-up), current light smokers (remained current light smokers at follow-up), current moderate smokers (remained current moderate smokers at follow-up) and current heavy smokers (remained current heavy smokers at follow-up).

Significance values are given in bold.

*Adjusted for age (years, continuous), sex, occupational grade (low; medium; high), depressive symptoms at baseline (no; yes), educational level (levels, continuous) and household income (€/month, continuous).

In current-smokers at baseline, high physical exertion decreased the odd of quitting (aOR 0.78, 95% CI 0.73–0.84) (Table 2). High physical exertion increased the odd of becoming heavy smokers (aOR 1.54, 95% CI 1.33–1.78). Dose-dependent relationships between physical exertion at work and tobacco use were found (*P*-trend < 0.001).

#### Cannabis use

High physical exertion at work was associated with an increased odd of using cannabis at least once per month at follow-up in participants who were not users for the last 12 months or more (aOR 1.31, 95% CI 1.03–1.66) (Table 3). When stratified by sex, this association was lost in men (Supplementary Table S8).

**Table 3:** Association between high physical exertion at work and cannabis use at 1-year of follow-up among employees in the CONSTANCES cohort study, 2012–2018 (odds ratios (ORs), and 95% confidence intervals, CI).

	N (%)	Unadjusted model	Fully-adjusted model*
		OR (95% CI)	OR (95% CI)
**Cannabis use**			
Relapse among ever-users at baseline	34,228		
No consumption in the past 12 months at follow-up	32,331 (94.5)	1.00	1.00
In the past 12 months, < 1/month	1558 (4.5)	0.90 (0.80–1.00)	0.93 (0.82–1.06)
In the past 12 months, ≥ 1/month	339 (1.0)	**1.67 (1.35–2.06)**	**1.31 (1.03–1.66)**

Significance values are given in bold.

*Adjusted for age (years, continuous), sex, occupational grade (low; medium; high), depressive symptoms at baseline (no; yes), educational level (levels, continuous) and household income (€/month, continuous).

#### Alcohol use

High physical exertion was not associated with alcohol use (Table 4).

**Table 4:** Association between high physical exertion at work and alcohol use at 1-year of follow-up among employees in the CONSTANCES cohort study, 2012–2018 (odds ratios (ORs), and 95% confidence intervals, CI).

	N (%)	Unadjusted model	Fully-adjusted model*
		OR (95% CI)	OR (95% CI)
**Alcohol use**			
Low risk	49,800 (66.0)	1.00	1.00
No use	15,762 (20.9)	**1.13 (1.09–1.17)**	1.01 (0.97–1.06)
At risk	9852 (13.1)	**1.09 (1.04–1.14)**	1.04 (0.98–1.10)

		*ß* (95% CI)	*ß* (95% CI)
Number of glasses/week	75,414	**0.02 (0.13; 0.16)**	0.11 (− 0.03; 0.26)

Alcohol use was defined as: low risk (1–27 drinks/week in men and 1–13 in women); no use and at risk (≥ 28 drinks/week in men and ≥ 14 in women).

Significance values are given in bold.

*Adjusted for age (years, continuous), sex, occupational grade (low; medium; high), depressive symptoms at baseline (no; yes), educational level (levels, continuous), household income (€/month, continuous) and baseline level of consumption.

#### Diet rich in sugar and fat

High perceived physical exertion was associated with an increased odd of consuming a diet rich in sugar and fat (aOR 1.06, 95% CI 1.01–1.11 and aOR 1.13, 95% CI 1.07–1.18, for the third and fourth quartiles compared to the first, respectively) (Table 5). Similar results while introducing the duration of follow-up as an additional covariable in the models, with no interaction between physical exertion and duration of follow-up.

**Table 5:** Association between high physical exertion at work and diet rich in sugar and fat at 5 years of follow-up among employees in the CONSTANCES cohort study, 2012–2018 (odds ratios (ORs), and 95% confidence intervals, CI).

	N (%)	Unadjusted model	Fully-adjusted model*
		OR (95% CI)	OR (95% CI)
**Diet rich in sugar and fat**			
First quartile	18,704 (24.8)	1.00	1.00
Second quartile	19,003 (25.2)	1.03 (0.98–1.07)	1.04 (0.99–1.09)
Third quartile	18,854 (25.0)	**1.07 (1.03–1.12)**	**1.06 (1.01–1.11)**
Fourth quartile	18,853 (25.0)	**1.12 (1.08–1.17)**	**1.13 (1.07–1.18)**
*P-*trend	** < 0.0001**		

Significance values are given in bold.

*Adjusted for age (years, continuous), sex, occupational grade (low; medium; high), depressive symptoms at baseline (no; yes), educational level (levels, continuous), household income (€/month, continuous) and baseline level of consumption.

When stratifying by age, no differences across age categories were found regarding the significant associations between, physical exertion at work and tobacco, cannabis and alcohol use and a diet rich in sugar and fat (Supplementary Table S9).

Similar findings were obtained in the non-imputed data (Supplementary Table S10).

### Supplementary analyses

Similar findings were obtained in the unadjusted and fully-adjusted Cox-proportional hazard models (Supplementary Table S11).

No significant interactions were observed between physical exertion and each of occupational grade, type of job contract and type of work (Supplementary Table S12) and no differences were found in the stratified analyses (Supplementary Tables S13–S15).

Lastly, similar findings were obtained in the fully-adjusted models where occupational grade educational level and household income were replaced by socioeconomic status (Supplementary Table S16).

## Discussion

This study examined the prospective association between physical exertion at work and tobacco, cannabis, alcohol use, and a diet rich in sugar and fat among employees from a large population-based cohort while taking into account sociodemographic factors and depressive symptoms. Overall, high physical exertion at work was positively associated with tobacco and cannabis use, as well as with the consumptions of a diet that is rich in sugar and fat. Regarding tobacco use, when exposed to high physical exertion at work, former smokers were more likely to relapse and current smokers were more likely to increase their consumption with dose-dependent relationships.

This study has some strengths. First, we used the RPE Borg scale which is a well-validated standardized tool to assess physical exertion at work^20^. Second, we had the necessary data to adjust the analyses for potential confounders, and sufficient power to run stratified analyses. This study has also some limitations. First, although such a large sample of population-based workers studies a large heterogeneity of different work settings, participants are not representative of the general population, even when randomly recruited. Thus, our results should be extrapolated with caution to other settings. Second, we cannot exclude the possibility of residual confounding since some contributing factors such as personality traits or other work factors like long-working hours, have not been measured. Third, even if we performed stratified analyses according to sociodemographic factors, we could not rule out that our associations concern only certain occupations. Fourth, the absence of quantities of fat and sugar intakes limited our ability to calculate energy intakes from these macronutrients and quantify their association with physical exertion at work. However, we do not believe this information may bias our finding as the frequency of consumption provides good information on nutrient intakes.

High physical exertion was associated with tobacco, cannabis use and diet rich in sugar and fat but not with alcohol use. Prior studies were mainly cross-sectional and none of them focused on physical exertion at work^23–27^. However, our findings are overall in accordance with prior findings showing increased risks of substance use in employees experiencing difficult working conditions (e.g., high job demand, work stress and work-related musculoskeletal disorders)^28–30^. We may hypothesize that work-related physical exertion could increase the need of more frequent breaks as well as close ties with peers to better cope with the intensity of work^31^. However, these situations may increase the likelihood of smoking^32^. Furthermore, physical exertion may promote sleep disorders^33^, and/or physical pain^34^, that one can try to alleviate by using cannabis. No associations were found with alcohol use. Even if this result has to be confirmed in future studies with longer duration of follow-up and measurement of alcohol use disorder, patterns of alcohol use mainly rely on other occupational factors (e.g., business lunches and machine driving) and may not be considered by employees as a good way to deal with physical exertion by seeking relaxation and rest rather than festive situations and disinhibition.

Two cross-sectional studies found that a detrimental work environment (measured by physical and biomechanical factors) was associated with obesity (BMI ≥ 30 kg/m^2^)^28,29^. Employees who are experiencing a tiring job could tend to cope with this situation by increasing or modifying their eating habits into comfortable foods (with a high fat and sugar content). Moreover, access to healthy foods may not always be available or easy at work, especially when exposed to difficult working conditions, making it thus harder to optimize food intake. Finally, our findings are overall in accordance with the propensity to use substance and to overconsume sugar and fat to cope with stressful life events^28,29,35–38^.

When stratified by age and sex, we did not find any differences across age categories. Hence, we believe that the probability of having less healthy individuals more likely to be unemployed than are healthy individuals to be low. Hence, the associations are unlikely to be explained by a “healthy worker effect” where older employees have better resilience regarding their work exposures^39^.

However, we found gender differences regarding tobacco and cannabis use. The lack of association in women while studying tobacco relapse is most likely the result of a reduction in statistical power. The lack of association in men while studying cannabis use needs further studies, especially by considering other potential confounders.

In conclusion, high perceived physical exertion at work was positively associated with tobacco and cannabis use and diet rich in sugar and fat. Regarding tobacco use, dose-dependent relationships were found, and when exposed to high physical exertion at work, former smokers were more likely to relapse whereas current smokers were less likely to quit. These associations should be considered when designing preventive strategies regarding poor health outcomes associated with physical exertion at work. For example, promoting other options to have a break than smoking, informing on healthy strategies to manage sleep disorders in the workplace. Furthermore, physical exertion, which can be shortly and easily assessed in occupational health, may be an indicator of the risk of unhealthy behaviors. Thus, employees exposed to such difficult working conditions should benefit from a standardized screening for substance use and deleterious eating behaviors, and to be referred to specialized care if needed. Since the present study did not find any substantial moderating effect of sociodemographic factors and depressive symptoms, these information and prevention strategies should be spread in all the exposed employees. Future studies should examine the benefits of such interventions on reducing the likelihood of unhealthy behaviors in employees.

## Supplementary Information

Supplementary Figure S1.

Supplementary Table S1.

Supplementary Table S2.

Supplementary Table S3.

Supplementary Table S4.

Supplementary Table S5.

Supplementary Table S6.

Supplementary Table S7.

Supplementary Table S8.

Supplementary Table S9.

Supplementary Table S10.

Supplementary Table S11.

Supplementary Table S12.

Supplementary Table S13.

Supplementary Table S14.

Supplementary Table S15.

Supplementary Table S16.

## Data Availability

Personal health data underlying the findings of our study are not publicly available due to legal reasons related to data privacy protection. However, the data are available upon request to all interested researchers after authorization of the French “Commission nationale de l’informatique et des libertés”. The CONSTANCES email address is contact@constances.fr.
