# Supplementary material for: Physical exertion at work and addictive behaviors: tobacco, cannabis, alcohol, sugar and fat consumption: longitudinal analyses in the CONSTANCES cohort
Source: Sci Rep. 2022 Jan 13;12:661. doi: 10.1038/s41598-021-04475-2 (PMC8758679; doi:10.1038/s41598-021-04475-2)
Supplement: Supplementary file 1 — Supplementary Figure S1. [file 41598_2021_4475_MOESM1_ESM.docx]

**Supplementary Fig S1**. The RPE Borg scale distribution by year of enrollment
