# Supplementary material for: Physical exertion at work and addictive behaviors: tobacco, cannabis, alcohol, sugar and fat consumption: longitudinal analyses in the CONSTANCES cohort
Source: Sci Rep. 2022 Jan 13;12:661. doi: 10.1038/s41598-021-04475-2 (PMC8758679; doi:10.1038/s41598-021-04475-2)
Supplement: Supplementary file 3 — Supplementary Table S2. [file 41598_2021_4475_MOESM3_ESM.docx]

**Supplementary Table S2.** The RPE Borg scale distribution by years of enrollment

|  | **Years** | | | | | |
| --- | --- | --- | --- | --- | --- | --- |
|  | **2012** | **2013** | **2014** | **2015** | **2016** | **2017** |
| **Borg scale** | **N(%)** | **N(%)** | **N(%)** | **N(%)** | **N(%)** | **N(%)** |
| **6** | 898 (17.0) | 2400 (19.9) | 3200 (20.2) | 3845 (19.6) | 4628 (20.5) | 4900 (19.4) |
| **7** | 817 (15.5) | 2000 (16.6) | 2300 (14.5) | 3100 (15.8) | 3426 (15.1) | 3712 (14.7) |
| **8** | 159 (3.0) | 330 (2.7) | 500 (3.2) | 570 (2.9) | 625 (2.8) | 828 (3.3) |
| **9** | 582 (11.0) | 1300 (10.8) | 1600 (10.1) | 1971 (10.1) | 2420 (10.7) | 2700 (10.7) |
| **10** | 127 (2.4) | 263 (2.2) | 300 (1.9) | 524 (2.7) | 555 (2.5) | 753 (3.0) |
| **11** | 990 (18.7) | 2060 (17.1) | 2401 (15.1) | 3210 (16.4) | 3502 (15.5) | 3525 (14.0) |
| **12** | 218 (4.1) | 562 (4.7) | 743 (4.7) | 1000 (5.1) | 1130 (5.0) | 1435 (5.7) |
| **13** | 630 (11.9) | 1417 (11.7) | 1990 (12.5) | 2327 (11.9) | 2546 (11.3) | 2970 (11.8) |
| **14** | 153 (2.9) | 360 (3.0) | 550 (3.5) | 643 (3.3) | 780 (3.4) | 906 (3.6) |
| **15** | 372 (7.0) | 719 (6.0) | 1300 (8.2) | 1226 (6.3) | 1732 (7.7) | 1698 (6.7) |
| **16** | 80 (1.5) | 188 (1.6) | 237 (1.5) | 360 (1.8) | 405 (1.8) | 548 (2.2) |
| **17** | 110 (2.1) | 273 (2.3) | 319 (2.0) | 410 (2.1) | 494 (2.2) | 560 (2.2) |
| **18** | 34 (0.6) | 55 (0.5) | 82 (0.5) | 103 (0.5) | 79 (0.3) | 161 (0.6) |
| **19** | 33 (0.6) | 48 (0.4) | 96 (0.6) | 79 (0.4) | 79 (0.3) | 178 (0.7) |
| **20** | 80 (1.5) | 100 (0.8) | 240 (1.5) | 210 (1.1) | 219 (1.0) | 324 (1.3) |
