# Supplementary material for: Physical exertion at work and addictive behaviors: tobacco, cannabis, alcohol, sugar and fat consumption: longitudinal analyses in the CONSTANCES cohort
Source: Sci Rep. 2022 Jan 13;12:661. doi: 10.1038/s41598-021-04475-2 (PMC8758679; doi:10.1038/s41598-021-04475-2)
Supplement: Supplementary file 5 — Supplementary Table S4. [file 41598_2021_4475_MOESM5_ESM.docx]

**Supplementary Table S4**. The principal component analysis of the socioeconomic status using the Varimax rotation.

| Items | Factor 1 (socioeconomic status) |
| --- | --- |
| Occupational grade | **0.88** |
| Household income | **0.73** |
| Educational level | **0.82** |
