# Supplementary material for: Physical exertion at work and addictive behaviors: tobacco, cannabis, alcohol, sugar and fat consumption: longitudinal analyses in the CONSTANCES cohort
Source: Sci Rep. 2022 Jan 13;12:661. doi: 10.1038/s41598-021-04475-2 (PMC8758679; doi:10.1038/s41598-021-04475-2)
Supplement: Supplementary file 6 — Supplementary Table S5. [file 41598_2021_4475_MOESM6_ESM.docx]

**Supplementary Table S5.** The correlation between educational level, occupational grade and household income.

| **Variables** | Educational level | Occupational grade | Household income |
| --- | --- | --- | --- |
| Educational level | 1.00 | 0.62 | 0.36 |
| Occupational grade | 0.62 | 1.00 | 0.48 |
| Household income | 0.36 | 0.48 | 1.00 |
