# Supplementary material for: Physical exertion at work and addictive behaviors: tobacco, cannabis, alcohol, sugar and fat consumption: longitudinal analyses in the CONSTANCES cohort
Source: Sci Rep. 2022 Jan 13;12:661. doi: 10.1038/s41598-021-04475-2 (PMC8758679; doi:10.1038/s41598-021-04475-2)
Supplement: Supplementary file 7 — Supplementary Table S6. [file 41598_2021_4475_MOESM7_ESM.docx]

**Supplementary Table S6**. The multicollinearity of occupational grade, household income and educational level in the fully-adjusted models for substance use and diet rich in sugar and fat.

| **Addictive behaviors** | **Tolerance** | **VIF** |
| --- | --- | --- |
| *Tobacco use* |  |  |
| *Relapse* |  |  |
| Occupational grade | 0.53 | 1.89 |
| Household income | 0.73 | 1.36 |
| Educational level | 0.57 | 1.77 |
| *Changing status among current-smokers* |  |  |
| Occupational grade | 0.54 | 1.85 |
| Household income | 0.73 | 1.35 |
| Educational level | 0.58 | 1.73 |
| *Changing status among ever-smokers* |  |  |
| Occupational grade | 0.53 | 1.05 |
| Household income | 0.72 | 1.39 |
| Educational level | 0.57 | 1.75 |
| *Number of cigarettes* |  |  |
| Occupational grade | 0.54 | 1.85 |
| Household income | 0.73 | 1.36 |
| Educational level | 0.57 | 1.75 |
| *Cannabis use* |  |  |
| Occupational grade | 0.52 | 1.91 |
| Household income | 0.73 | 1.37 |
| Educational level | 0.57 | 1.76 |
| *Alcohol use* |  |  |
| Occupational grade | 0.51 | 1.97 |
| Household income | 0.70 | 1.42 |
| Educational level | 0.54 | 1.84 |
| *Diet rich in sugar and fat* |  |  |
| Occupational grade | 0.51 | 1.96 |
| Household income | 0.70 | 1.43 |
| Educational level | 0.54 | 1.84 |
