# Supplementary material for: Physical exertion at work and addictive behaviors: tobacco, cannabis, alcohol, sugar and fat consumption: longitudinal analyses in the CONSTANCES cohort
Source: Sci Rep. 2022 Jan 13;12:661. doi: 10.1038/s41598-021-04475-2 (PMC8758679; doi:10.1038/s41598-021-04475-2)
Supplement: Supplementary file 9 — Supplementary Table S8. [file 41598_2021_4475_MOESM9_ESM.docx]

**Supplementary Table S8.** Association between high physical exertion at work and addictive behaviors at one-year of follow-up according to sex among employees in the CONSTANCES cohort study, 2012-2018 (odds ratios (ORs), and 95% confidence intervals, CI).

|  | **Women** | | |  | **Men** | | |
| --- | --- | --- | --- | --- | --- | --- | --- |
|  |  | **Unadjusted model** | **Fully-adjusted model*** |  |  | **Unadjusted model** | **Fully-adjusted model*** |
| **Addictive behaviors** | **N (%)** | **OR (95% CI)** | **OR (95% CI)** |  | **N (%)** | **OR (95% CI)** | **OR (95% CI)** |
| **Tobacco use** |  |  |  |  |  |  |  |
| *Relapse of tobacco use among ex-smokers at baseline* | 15,464 |  |  |  | 15,452 |  |  |
| No | 12,735 (82.4) | 1.00 | 1.00 |  | 12,483 (80.8) | 1.00 | 1.00 |
| Yes | 2,729 (17.6) | **1.24 (1.13-1.35)** | 1.12 (0.94-1.33) |  | 2,969 (19.2) | **1.48 (1.37-1.61)** | **1.12 (1.01-1.23)** |
|  |  |  |  |  |  |  |  |
| *Changing status among current smokers at baseline* | 10,128 |  |  |  | 9,950 |  |  |
| Ex-smoker | 2,982 (29.4) | 1.00 | 1.00 |  | 2,805 (28.2) | 1.00 | 1.00 |
| Current light smoker | 4,465 (44.1) | **1.40 (1.26-1.54)** | **1.23 (1.10-1.37)** |  | 3,939 (39.6) | **1.73 (1.56-1.91)** | **1.15 (1.02-1.30)** |
| Current moderate Smoker | 2,260 (22.3) | **1.73 (1.54-1.94)** | **1.30 (1.15-1.47)** |  | 2,491 (25.0) | **2.57 (2.30-2.88)** | **1.33 (1.16-1.52)** |
| Current heavy smoker | 421 (4.2) | **2.02 (1.64-2.48)** | **1.57 (1.26-1.97)** |  | 713 (7.2) | **2.74 (2.31-3.24)** | **1.45 (1.19-1.77)** |
| *P-trend* | **<0.0001** |  |  |  | **<0.0001** |  |  |
|  |  |  |  |  |  |  |  |
| *Changing status among ever-smokers at baseline* | 25,592 |  |  |  | 25,402 |  |  |
| Smoker at baseline and remained smoker at follow-up | 7,146 (27.9) | 1.00 | 1.00 |  | 7,145 (28.2) | 1.00 | 1.00 |
| Smoker at baseline and stopped at follow-up | 2,982 (11.6) | **0.65 (0.60-0.72)** | **0.79 (0.71-0.87)** |  | 2,805 (11.0) | **0.48 (0.44-0.53)** | **0.81 (0.72-0.90)** |
| Ex-smoker at baseline and stopped at follow-up | 12,735 (49.8) | **0.67 (0.63-0.71)** | **0.86 (0.80-0.92)** |  | 12,483 (49.1) | **0.55 (0.52-0.58)** | **0.86 (0.80-0.92)** |
| Ex-smoker at baseline and started smoking at follow-up | 2,729 (10.7) | **0.83 (0.75-0.91)** | 0.93 (0.84-1.02) |  | 2,969 (11.7) | **0.81 (0.75-0.89)** | 0.94 (0.85-1.04) |
| *P-trend* | **<0.0001** |  |  |  | **<0.0001** |  |  |
|  |  |  |  |  |  |  |  |
|  |  | ***ß* (95%CI)** | ***ß* (95%CI)** |  |  | ***ß* (95%CI)** | ***ß* (95%CI)** |
| *Number of cigarettes/day among current smokers at baseline* | 10,128 | 0.07 (-0.14;0.28) | **0.33 (0.13;0.52)** |  | 9,950 | 0.02 (-0.22;0.27) | **0.32 (0.08;0.56)** |
|  |  |  |  |  |  |  |  |
| **Cannabis use** |  | **OR (95% CI)** | **OR (95% CI)** |  |  | **OR (95% CI)** | **OR (95% CI)** |
| *Relapse among ever-users at baseline* | 16,304 |  |  |  | 17,924 |  |  |
| No consumption in the past 12 months at follow-up | 15,514 (95.1) | 1.00 | 1.00 |  | 16,817 (93.8) | 1.00 | 1.00 |
| In the past 12 months, <1/month | 628 (3.9) | 0.93 (0.77-1.11) | 0.95 (0.79-1.16) |  | 930 (5.2) | **0.86 (0.74-0.98)** | 0.92 (0.78-1.09) |
| In the past 12 months, ≥1/month | 162 (1.0) | **1.76 (1.29-2.40)** | **1.56 (1.12-2.18)** |  | 177 (1.0) | **1.59 (1.19-2.13)** | 1.05 (0.75-1.47) |
|  |  |  |  |  |  |  |  |
|  |  | **OR (95% CI)** | **OR (95% CI)** |  |  | **OR (95% CI)** | **OR (95% CI)** |
| **Alcohol use** | 39,767 |  |  |  | 35,647 |  |  |
| Low risk | 22,246 (55.9) | 1.00 | 1.00 |  | 27,554 (77.3) | 1.00 | 1.00 |
| No use | 10,785 (27.1) | **1.12 (1.07-1.18)** | 1.00 (0.95-1.06) |  | 4,977 (14.0) | **1.20 (1.13-1.28)** | 1.03 (0.95-1.11) |
| At risk | 6,736 (17.0) | 1.02 (0.96-1.08) | 1.01 (0.95-1.08) |  | 3,116 (8.7) | **1.30 (1.21-1.41)** | 1.04 (0.94-1.14) |
|  |  |  |  |  |  |  |  |
|  |  | ***ß* (95%CI)** | ***ß* (95%CI)** |  |  | ***ß* (95%CI)** | ***ß* (95%CI)** |
| *Number of glasses/week* | 39,767 | **0.18 (0.01;0.35)** | 0.04 (-0.11;0.19) |  | 35,647 | -0.19 (-0.44;0.06) | 0.12 (-0.15;0.38) |
|  |  |  |  |  | 8520 |  |  |
| **Diet rich in sugar and fat** | 39,767 | **OR (95% CI)** | **OR (95% CI)** |  | 35,647 | **OR (95% CI)** | **OR (95% CI)** |
| First quartile | 9,861 (24.8) | 1.00 | 1.00 |  | 8,842 (24.8) | 1.00 | 1.00 |
| Second quartile | 10,022 (25.2) | 1.02 (0.96-1.08) | 1.04 (0.98-1.11) |  | 8,981 (25.2) | 0.99 (0.93-1.05) | 1.02 (0.95-1.10) |
| Third quartile | 9,942 (25.0) | **1.05 (1.01-1.11)** | **1.07 (1.01-1.14)** |  | 8,912 (25.0) | **1.06 (1.01-1.13)** | **1.06 (1.02-1.14)** |
| Fourth quartile | 9,942 (25.0) | **1.10 (1.05-1.18)** | **1.12 (1.05-1.19)** |  | 8,912 (25.0) | **1.10 (1.02-1.12)** | **1.10 (1.02-1.18)** |
| *P-trend* | **<0.0001** |  |  |  | **<0.0001** |  |  |

| *Adjusted for age (years, continuous), occupational grade (low; medium; high), depressive symptoms at baseline (no; yes), educational level (levels, continuous), household income (€/month, continuous) and baseline level of consumption. |
| --- |
| Categories of current smokers were defined as: light smokers (<10 cigarettes/day), moderate smokers (10-18 cigarettes/day) and heavy smokers (>19 cigarettes/day). |
| Relapse was defined as: no (remained non-smokers at follow-up) and yes (became current smokers at follow-up). |
| Changing status among current smokers was defined as ex-smokers (stopped smoking at follow-up), current light smokers (remained current light smokers at follow-up), current moderate smokers (remained current moderate smokers at follow-up) and current heavy smokers (remained current heavy smokers at follow-up).  Alcohol use was defined as: low risk (1-27 drinks/week in men and 1-13 i\n women); no use and at risk (≥28 drinks/week in men and ≥14 in women). |
