# Supplementary material for: Physical exertion at work and addictive behaviors: tobacco, cannabis, alcohol, sugar and fat consumption: longitudinal analyses in the CONSTANCES cohort
Source: Sci Rep. 2022 Jan 13;12:661. doi: 10.1038/s41598-021-04475-2 (PMC8758679; doi:10.1038/s41598-021-04475-2)
Supplement: Supplementary file 10 — Supplementary Table S9. [file 41598_2021_4475_MOESM10_ESM.docx]

**Supplementary Table S9.** Association between high physical exertion and addictive behaviors according to age.

|  | **Age** | | | | | | | | | | | | | | | |
| --- | --- | --- | --- | --- | --- | --- | --- | --- | --- | --- | --- | --- | --- | --- | --- | --- |
|  | **18-29** | | | **30-39** | | | **40-49** | | | **50-59** | | | **60+** | | | |
|  |  | **Unadjusted** | **Fully-adjusted *** |  | **Unadjusted** | **Fully-adjusted *** |  | **Unadjusted** | **Fully-adjusted *** |  | **Unadjusted** | **Fully-adjusted *** |  | **Unadjusted** | **Fully-adjusted *** |  |
| **Addictive behaviors** | **N (%)** | **OR (95% CI)** | **OR (95% CI)** | **N (%)** | **OR (95% CI)** | **OR (95% CI)** | **N (%)** | **OR (95% CI)** | **OR (95% CI)** | **N (%)** | **OR (95% CI)** | **OR (95% CI)** | **N (%)** | **OR (95% CI)** | **OR (95% CI)** |  |
| **Tobacco use** |  |  |  |  |  |  |  |  |  |  |  |  |  |  |  |  |
| Relapse of tobacco use among ex-smokers at baseline | 2 001 |  |  | 6 523 |  |  | 9 611 |  |  | 10206 |  |  | 2 575 |  |  |  |
| No | 1 419 (70.1) | 1.00 | 1.00 | 5 080 (77.9) | 1.00 | 1.00 | 7 804 (81.2) | 1.00 | 1.00 | 8 688 (85.1) | 1.00 | 1.00 | 2 227 (86.5) | 1.00 | 1.00 |  |
| Yes | 582 (29.1) | 1.19 (0.97-1.45) | 0.95 (0.70-1.28) | 1 443 (22.1) | **1.32 (1.17-1.50)** | 1.18 (0.93-1.49) | 1 807 (18.8) | **1.30 (1.17-1.45)** | 1.08 (0.96-1.21) | 1 518 (14.9) | **1.48 (1.32-1.65)** | **1.16 (1.02-1.33)** | 348 (13.5) | **1.77 (1.39-2.24)** | 1.32 (1.01-1.75) |  |
|  |  |  |  |  |  |  |  |  |  |  |  |  |  |  |  |  |
| Changing status among current smokers at baseline | 3 629 |  |  | 5 942 |  |  | 5 742 |  |  | 4 100 |  |  | 665 |  |  |  |
| Ex-smoker | 1 031 (28.4) | 1.00 | 1.00 | 1 882 (31.7) | 1.00 | 1.00 | 1 568 (27.3) | 1.00 | 1.00 | 1 102 (27.0) | 1.00 | 1.00 | 202 (30.4) | 1.00 | 1.00 |  |
| Current light smoker | 1 739 (47.9) | **1.67 (1.42-1.96)** | **1.26 (1.05-1.51)** | 2 507 (42.2) | **1.57 (1.38-1.79)** | 1.17 (0.96-1.35) | 2 277 (39.6) | **1.48 (1.29-1.70)** | 1.16 (0.98-1.35) | 1 613 (39.3) | **1.51 (1.28-1.71)** | **1.32 (1.11-1.57)** | 271 (40.8) | 0.93 (0.62-1.39) | 0.81 (0.53-1.26) |  |
| Current moderate Smoker | 762 (21.0) | **2.46 (2.02-2.98)** | **1.36 (1.09-1.69)** | 1 311 (22.1) | **2.07 (1.79-2.41)** | 1.18 (0.98-1.39) | 1 487 (25.9) | **2.13 (1.84-2.48)** | **1.32 (1.12-1.56)** | 1 059 (25.8) | **2.11 (1.77-2.51)** | **1.51 (1.25-1.83)** | 133 (19.9) | 1.46 (0.92-2.32) | 1.03 (0.62-1.71) |  |
| Current heavy smoker | 97 (2.7) | **3.65 (2.36-5.65)** | **1.87 (1.15-3.05)** | 242 (4.0) | **3.05 (2.32-3.99)** | **1.78 (1.31-2.43)** | 410 (7.2) | **2.53 (2.02-3.16)** | **1.46 (1.14-1.89)** | 326 (8.0) | **1.93 (1.50-2.48)** | **1.30 (1.01-1.73)** | 59 (8.2) | **1.98 (1.02-3.63)** | 1.42 (0.74-2.75) |  |
| *P-trend* | **<0.0001** |  |  | **<0.0001** |  |  | **<0.0001** |  |  |  |  |  | **<0.0001** |  |  |  |
|  |  |  |  |  |  |  |  |  |  |  |  |  |  |  |  |  |
| Changing status among ever-smokers at baseline | 5 630 |  |  | 12 465 |  |  | 15353 |  |  | 14306 |  |  | 3 240 |  |  |  |
| Smoker at baseline and remained smoker at follow-up | 2 597 (46.1) | 1.00 | 1.00 | 4 060 (32.6) | 1.00 | 1.00 | 4 174 (27.2) | 1.00 | 1.00 | 2 998 (21.0) | 1.00 | 1.00 | 463 (14.4) | 1.00 | 1.00 |  |
| Smoker at baseline and stopped at follow-up | 1 031 (18.3) | **0.52 (0.45-0.60)** | **0.76 (0.64-0.90)** | 1 882 (15.1) | **0.56 (0.49-0.63)** | **0.82 (0.72-0.94)** | 1 568 (10.2) | **0.56 (0.49-0.64)** | **0.79 (0.69-0.91)** | 1 102 (7.7) | **0.57 (0.49-0.66)** | **0.73 (0.62-0.86)** | 202 (6.2) | 0.85 (0.59-1.22) | 1.07 (0.72-1.58) |  |
| Ex-smoker at baseline and stopped at follow-up | 1 419 (25.2) | **0.59 (0.52-0.68)** | 0.88 (0.76-1.03) | 5 080 (40.7) | **0.57 (0.53-0.63)** | **0.84 (0.76-0.93)** | 7 804 (50.8) | **0.67 (0.62-0.73)** | 0.94 (0.86-1.02) | 8 688 (60.7) | **0.60 (0.57-0.66)** | **0.79 (0.72-0.87)** | 2 227 (68.7) | **0.76 (0.61-0.94)** | 0.95 (0.75-1.20) |  |
| Ex-smoker at baseline and started smoking at follow-up | 582 (10.4) | **0.71 (0.59-0.85)** | 0.82 (0.67-1.01) | 1 443 (11.6) | **0.76 (0.70-0.86)** | 0.92 (0.80-1.06) | 1 807 (11.8) | **0.88 (0.78-0.98)** | 1.00 (0.88-1.13) | 1 518 (10.6) | **0.89 (0.79-0.97)** | 0.93 (0.81-1.07) | 348 (10.7) | **1.34 (1.01-1.80)** | 1.22 (0.89-1.67) |  |
| *P-trend* | **<0.0001** |  |  | **<0.0001** |  |  | **<0.0001** |  |  | **<0.0001** |  |  |  |  |  |  |
|  |  |  |  |  |  |  |  |  |  |  |  |  |  |  |  |  |
| Number of cigarettes/day among current smokers at baseline |  |  |  |  |  |  |  |  |  |  |  |  |  |  |  |  |
| Absolute, continuous | 3 629 | **1.94 (1.59;2.30)** | **0.38 (0.06;0.69)** | 5 942 | **1.76 (1.46-2.07)** | 0.26 (-0.01;0.53) | 5 742 | **2.09 (1.74-2.45)** | 0.22 (-0.08;0.53) | 4 100 | **1.65 (1.23;2.07)** | **0.56 (0.20;0.92)** | 665 | 1.34 (-0.01;2.50) | 0.01 (-1.00;1.01) |  |
| Baseline and follow-up difference, continuous | 3 629 | -0.08 (-0.32;0.33) | **0.38 (0.06;0.69)** | 5 942 | -0.06 (-0.33;0.21) | 0.26 (-0.01;0.53) | 5 742 | **-0.34 (-0.06;-0.03)** | 0.22 (-0.08;0.53) | 4 100 | 0.38 (-0.01;0.78) | **0.56 (0.20;0.92)** | 665 | -0.54 (-1.64;0.55) | 0.01 (-1.00;1.01) |  |
|  |  |  |  |  |  |  |  |  |  |  |  |  |  |  |  |  |
| **Cannabis** |  |  |  |  |  |  |  |  |  |  |  |  |  |  |  |  |
| *Relapse among ever-users at baseline* | 5 228 |  |  | 11 461 |  |  | 10466 |  |  | 6 078 |  |  | 995 |  |  |  |
| No consumption in the past 12 months at follow-up | 4 746 (90.8) | 1.00 | 1.00 | 10 748 (93.8) | 1.00 | 1.00 | 10 006 (95.5) | 1.00 | 1.00 | 5 870 (96.6) | 1.00 | 1.00 | 965 (97.2) | 1.00 | 1.00 |  |
| In the past 12 months, <1/month | 416 (7.9) | 0.88 (0.71-1.09) | 1.01 (0.79-1.28) | 591 (5.1) | 0.82 (0.68-1.01) | 0.87 (0.70-1.08) | 363 (3.5) | 0.90 (0.72-1.14) | 0.93 (0.72-1.20) | 163 (2.7) | 0.83 (0.58-1.16) | 0.90 (0.61-1.31) | 21 (2.0) | 1.37 (0.52-3.57) | 1.00 (0.36-2.81) |  |
| In the past 12 months, ≥1/month | 66 (1.3) | **1.79 (1.11-2.88)** | 1.65 (0.96-2.82) | 122 (1.1) | **1.93 (1.37-2.73)** | **1.55 (1.05-2.29)** | 97 (1.0) | 1.03 (0.66-1.60) | 0.83 (0.51-1.34) | 45 (0.7) | **2.44 (1.37-4.33)** | **1.92 (1.01-3.66)** | 9 (0.8) | 0.98 (0.20-4.73) | 0.64 (0.12-3.44) |  |
|  |  |  |  |  |  |  |  |  |  |  |  |  |  |  |  |  |
| **Alcohol intake, number of glasses/week** |  | ***ß* (95%CI)** | ***ß* (95%CI)** |  | ***ß* (95%CI)** | ***ß* (95%CI)** |  | ***ß* (95%CI)** | ***ß* (95%CI)** |  | ***ß* (95%CI)** | ***ß* (95%CI)** |  | ***ß* (95%CI)** | ***ß* (95%CI)** |  |
| Absolute, continuous | 9 481 | 0.39 (-0.05;0.83) | 0.20 (-0.21;0.62) | 17 962 | **0.49 (0.17;0.81)** | 0.15 (-0.14;0.45) | 22 544 | **0.66 (0.37;0.95)** | -0.13 (-0.12;0.39) | 20 799 | **0.38 (0.05;0.71)** | 0.07 (-0.22;0.35) | 4 628 | 0 (-0.76;0.77) | 0.26 (-0.39;0.90) |  |
| Baseline and follow-up difference, continuous | 9 481 | 0.08 (-0.15;0.41) | 0.20 (-0.21;0.62) | 17 962 | 0.22 (-0.08;0.52) | 0.15 (-0.14;0.45) | 22 544 | 0.01 (-0.25;0.26) | -0.13 (-0.12;0.39) | 20 799 | -0.11 (-0.39;0.17) | 0.07 (-0.22;0.35) | 4 628 | -0.07 (-0.70;0.56) | 0.26 (-0.39;0.90) |  |
|  |  |  |  |  |  |  |  |  |  |  |  |  |  |  |  |  |
| **Sugar and fat intake** |  | ***ß* (95%CI)** | ***ß* (95%CI)** |  | ***ß* (95%CI)** | ***ß* (95%CI)** |  | ***ß* (95%CI)** | ***ß* (95%CI)** |  | ***ß* (95%CI)** | ***ß* (95%CI)** |  | ***ß* (95%CI)** | ***ß* (95%CI)** |  |
| Continuous | 9 481 | **0.10 (0.06;0.14)** | **0.06 (0.02;0.10)** | 17 962 | **0.06 (0.03;0.09)** | **0.05 (0.02;0.08)** | 22 544 | **0.04 (0.01;0.07)** | **0.04 (0.01;0.07)** | 20 799 | **0.04 (0.01;0.07)** | **0.03 (0.01;0.06)** | 4 628 | -0.01 (-0.07;0.05) | 0.02 (-0.04;0.09) |  |
|  |  |  |  |  |  |  |  |  |  |  |  |  |  |  |  |  |
|  |  |  |  |  |  |  |  |  |  |  |  |  |  |  |  |  |
| First quartile | 2 409 (25.4) | 1.00 | 1.00 | 4 553 (25.4) | 1.00 | 1.00 | 5 762 (25.5) | 1.00 | 1.00 | 5 182 (24.9) | 1.00 | 1.00 | 1 153 (24.9) | 1.00 | 1.00 |  |
| Second quartile | 2 286 (24.1) | 1.02 (0.91-1.15) | 1.00 (0.88-1.14) | 4 428 (24.6) | 1.00 (0.91-1.09) | 1.03 (0.93-1.14) | 5 473 (24.3) | 1.01 (0.94-1.10) | 1.05 (0.96-1.14) | 5 258 (25.3) | 1.05 (0.97-1.14) | 1.03 (0.94-1.12) | 1 166 (25.2) | 0.96 (0.80-1.15) | 1.06 (0.87-1.28) |  |
| Third quartile | 2 417 (25.5) | **1.10 (1.03-1.25)** | 1.00 (0.88-1.14) | 4 595 (25.6) | 1.07 (0.98-1.17) | 1.06 (0.96-1.18) | 5 673 (25.2) | 1.05 (0.97-1.14) | 1.04 (0.95-1.14) | 5 177 (24.9) | 1.04 (0.96-1.13) | 1.00 (0.97-1.10) | 1 145 (24.8) | 1.01 (0.85-1.21) | 1.09 (0.89-1.32) |  |
| Fourth quartile | 2 370 (25.0) | **1.28 (1.14-1.44)** | **1.16 (1.02-1.33)** | 4 386 (24.4) | **1.12 (1.03-1.23)** | **1.13 (1.02-1.25)** | 5 636 (25.0) | **1.09 (1.01-1.18)** | **1.10 (1.01-1.20)** | 5 182 (24.9) | **1.09 (1.02-1.18)** | 1.06 (0.97-1.16) | 1 164 (25.1) | 1.01 (0.85-1.21) | 1.15 (0.95-1.41) |  |
| *P-trend* | **<0.0001** |  |  | **<0.0001** | |  | **<0.0001** |  |  |  |  |  | 0.26 |  |  |  |

| *Adjusted for sex, occupational grade (low; medium; high), depressive symptoms at baseline (no; yes), educational level (levels, continuous), household income (€/month, continuous) and baseline level of consumption. |
| --- |
| Categories of current smokers were defined as: light smokers (<10 cigarettes/day), moderate smokers (10-18 cigarettes/day) and heavy smokers (>19 cigarettes/day). |
| Relapse was defined as: no (remained non-smokers at follow-up) and yes (became current smokers at follow-up). |
| Changing status among current smokers was defined as ex-smokers (stopped smoking at follow-up), current light smokers (remained current light smokers at follow-up), current moderate smokers (remained current moderate smokers at follow-up) and current heavy smokers (remained current heavy smokers at follow-up). |
