# Supplementary material for: Physical exertion at work and addictive behaviors: tobacco, cannabis, alcohol, sugar and fat consumption: longitudinal analyses in the CONSTANCES cohort
Source: Sci Rep. 2022 Jan 13;12:661. doi: 10.1038/s41598-021-04475-2 (PMC8758679; doi:10.1038/s41598-021-04475-2)
Supplement: Supplementary file 11 — Supplementary Table S10. [file 41598_2021_4475_MOESM11_ESM.docx]

**Supplementary Table S10.** Association between high physical exertion at work and addictive behaviors at one-year of follow-up among employees in the non-imputed data of the CONSTANCES cohort study, 2012-2018 (odds ratios (ORs), and 95% confidence intervals, CI).

|  | **Unadjusted model** | **Fully-adjusted model*** |  |
| --- | --- | --- | --- |
| **Addictive behaviors** | **OR (95% CI)** | **OR (95% CI)** |  |
| **Tobacco use** |  |  |  |
| *Relapse of tobacco use among ex-smokers at baseline* |  |  |  |
| No | 1.00 | 1.00 |  |
| Yes | **1.14 (1.01-1.29)** | **1.10 (1.01-1.20)** |  |
|  |  |  |  |
| *Changing status among current smokers at baseline* |  |  |  |
| Ex-smoker | 1.00 | 1.00 |  |
| Current light smoker | **1.21 (1.11-1.32)** | **1.10 (1.01-1.24)** |  |
| Current moderate Smoker | **1.83 (1.67-2.01)** | **1.25 (1.12-1.40)** |  |
| Current heavy smoker | **2.24 (1.95-2.58)** | **1.51 (1.27-1.80)** |  |
| *P-trend* | **<0.0001** |  |  |
|  |  |  |  |
| *Changing status among ever-smokers at baseline* |  |  |  |
| Smoker at baseline and remained smoker at follow-up | 1.00 | 1.00 |  |
| Smoker at baseline and stopped at follow-up | **0.65 (0.61-0.70)** | **0.83 (0.76-0.90)** |  |
| Ex-smoker at baseline and stopped at follow-up | **0.68 (0.65-0.72)** | **0.89 (0.83-0.95)** |  |
| Ex-smoker at baseline and started smoking at follow-up | **0.78 (0.68-0.89)** | 0.91 (0.79-1.07) |  |
| *P-trend* | **<0.0001** |  |  |
|  |  |  |  |
|  | ***ß* (95%CI)** | ***ß* (95%CI)** |  |
| *Number of cigarettes/day among current smokers at baseline* | 0 (-0.21;0.21) | **0.21 (0.01;0.43)** |  |
|  |  |  |  |
| **Cannabis use** | **OR (95% CI)** | **OR (95% CI)** |  |
| *Relapse among ever-users at baseline* |  |  |  |
| No consumption in the past 12 months at follow-up | 1.00 | 1.00 |  |
| In the past 12 months, <1/month | 1.02 (0.87-1.19) | 0.99 (0.82-1.19) |  |
| In the past 12 months, ≥1/month | **1.56 (1.10-2.21)** | **1.40 (1.01-2.11)** |  |
|  |  |  |  |
| **Alcohol use** |  |  |  |
| Low risk | 1.00 | 1.00 |  |
| No use | **1.57 (1.52-1.62)** | 1.13 (0.99-1.18) |  |
| At risk | **1.09 (1.03-1.16)** | 1.05 (0.97-1.13) |  |
|  |  |  |  |
|  | ***ß* (95%CI)** | ***ß* (95%CI)** |  |
| *Number of glasses/week* | -0.11 (-0.30;0.07) | 0.04 (-0.24;0.16) |  |
|  |  |  |  |
| **Sugar and fat consumption** | **OR (95% CI)** | **OR (95% CI)** |  |
| First quartile | 1.00 | 1.00 |  |
| Second quartile | 1.03 (0.96-1.10) | 1.09 (0.96-1.24) |  |
| Third quartile | 1.05 (0.98-1.12) | **1.15 (1.00-1.32)** |  |
| Fourth quartile | **1.13 (1.06-1.21)** | **1.20 (1.03-1.40)** |  |
| *P-trend* | **0.003** | **0.001** |  |
| *Adjusted for age (years, continuous), sex, occupational grade (low; medium; high), depressive symptoms at baseline (no; yes), educational level (levels, continuous), household income (€/month, continuous) and baseline level of consumption. | | | |
| Categories of current smokers were defined as: light smokers (<10 cigarettes/day), moderate smokers (10-18 cigarettes/day) and heavy smokers (>19 cigarettes/day). | | | |
| Relapse was defined as: no (remained non-smokers at follow-up) and yes (became current smokers at follow-up). | | | |
| Changing status among current smokers was defined as ex-smokers (stopped smoking at follow-up), current light smokers (remained current light smokers at follow-up), current moderate smokers (remained current moderate smokers at follow-up) and current heavy smokers (remained current heavy smokers at follow-up).  Alcohol use was defined as: low risk (1-27 drinks/week in men and 1-13 in women); no use and at risk (≥28 drinks/week in men and ≥14 in women). | | | |
