# Supplementary material for: Physical exertion at work and addictive behaviors: tobacco, cannabis, alcohol, sugar and fat consumption: longitudinal analyses in the CONSTANCES cohort
Source: Sci Rep. 2022 Jan 13;12:661. doi: 10.1038/s41598-021-04475-2 (PMC8758679; doi:10.1038/s41598-021-04475-2)
Supplement: Supplementary file 12 — Supplementary Table S11. [file 41598_2021_4475_MOESM12_ESM.docx]

**Supplementary Table S11.** Association between high physical exertion and addictive behaviors (hazard ratios (HRs), 95% confidence intervals, CI).

|  |  | **Unadjusted model** | **Fully-adjusted model*** |  |
| --- | --- | --- | --- | --- |
| **Addictive behaviors** | **N (%)** | **HR (95% CI)** | **HR (95% CI)** |  |
| **Tobacco use** |  |  |  |  |
| *Relapse of tobacco use among ex-smokers at baseline* | 30,916 |  |  |  |
| No | 25,218 (81.6) | 1.00 | 1.00 |  |
| Yes | 5,698 (18.4) | **1.33 (1.24-1.42)** | **1.09 (1.01-1.17)** |  |
|  |  |  |  |  |
| *Changing status among current smokers at baseline* | 20,078 |  |  |  |
| Ex-smoker | 5,787 (28.8) | 1.00 | 1.00 |  |
| Current light smoker | 8,406 (41.9) | 0.99 (0.94-1.04) | 0.97 (0.92-1.03) |  |
| Current moderate Smoker | 4,751 (23.7) | **1.40 (1.31-1.50)** | **1.09 (1.01-1.17)** |  |
| Current heavy smoker | 1,134 (5.6) | **1.63 (1.43-1.86)** | **1.25 (1.08-1.45)** |  |
| *P-trend* | **<0.0001** |  |  |  |
|  |  |  |  |  |
| *Changing status among ever-smokers at baseline* | 50,994 |  |  |  |
| Smoker at baseline and remained smoker at follow-up | 14,291 (28.0) | 1.00 | 1.00 |  |
| Smoker at baseline and stopped at follow-up | 5,787 (11.3) | **0.79 (0.74-0.84)** | **0.86 (0.80-0.93)** |  |
| Ex-smoker at baseline and stopped at follow-up | 25,218 (49.5) | **0.83 (0.81-0.86)** | **0.93 (0.90-0.96)** |  |
| Ex-smoker at baseline and started smoking at follow-up | 5,698 (11.2) | **1.17 (1.10-1.25)** | 1.04 (0.97-1.12) |  |
| *P-trend* | **<0.0001** |  |  |  |
|  |  |  |  |  |
| **Cannabis use** |  |  |  |  |
| *Relapse among ever-users at baseline* | 34,228 |  |  |  |
| No consumption in the past 12 months at follow-up | 32,331 (94.5) | 1.00 | 1.00 |  |
| In the past 12 months, <1/month | 1,558 (4.5) | 0.86 (0.77-0.97) | 0.91 (0.81-1.03) |  |
| In the past 12 months, ≥1/month | 339 (1.0) | **1.61 (1.30-1.98)** | **1.27 (1.00-1.60)** |  |
|  |  |  |  |  |
| **Alcohol use** |  |  |  |  |
| Low risk | 49,800 (66.0) | 1.00 | 1.00 |  |
| No use | 15,762 (20.9) | **1.08 (1.05-1.12)** | 0.98 (0.94-1.01) |  |
| At risk | 9,852 (13.1) | **1.05 (1.01-1.09)** | 1.02 (0.97-1.06) |  |
|  |  |  |  |  |
| **Diet rich in sugar and fat** |  | **HR (95% CI)** | **HR (95% CI)** |  |
| First quartile | 18,704 (24.8) | 1.00 | 1.00 |  |
| Second quartile | 19,003 (25.2) | 0.97 (0.94-1.01) | 0.97 (0.93-1.01) |  |
| Third quartile | 18,854 (25.0) | 1.01 (0.98-1.04) | 0.98 (0.95-1.01) |  |
| Fourth quartile | 18,853 (25.0) | **1.06 (10.3-1.09)** | **1.03 (1.01-1.07)** |  |
| *P-trend* | **<0.0001** |  |  |  |
| *Adjusted for age (years, continuous), sex, occupational grade (low; medium; high), depressive symptoms at baseline (no; yes), educational level (levels, continuous), household income (€/month, continuous) and baseline level of consumption. | | | | |
| Categories of current smokers were defined as: light smokers (<10 cigarettes/day), moderate smokers (10-18 cigarettes/day) and heavy smokers (>19 cigarettes/day). | | | | |
| Relapse was defined as: no (remained non-smokers at follow-up) and yes (became current smokers at follow-up). | | | | |
| Changing status among current smokers was defined as ex-smokers (stopped smoking at follow-up), current light smokers (remained current light smokers at follow-up), current moderate smokers (remained current moderate smokers at follow-up) and current heavy smokers (remained current heavy smokers at follow-up).  Alcohol use was defined as: low risk (1-27 drinks/week in men and 1-13 in women); no use and at risk (≥28 drinks/week in men and ≥14 in women). | | | | |
