# Supplementary material for: Physical exertion at work and addictive behaviors: tobacco, cannabis, alcohol, sugar and fat consumption: longitudinal analyses in the CONSTANCES cohort
Source: Sci Rep. 2022 Jan 13;12:661. doi: 10.1038/s41598-021-04475-2 (PMC8758679; doi:10.1038/s41598-021-04475-2)
Supplement: Supplementary file 13 — Supplementary Table S12. [file 41598_2021_4475_MOESM13_ESM.docx]

**Supplemental Table S12.** Interactions between each of occupational grade, type of work and type of job contract and physical exertion at work while studying their effects on addictive behaviors at one-year of follow-up among employees in the CONSTANCES cohort study, 2012-2018 (P-values).

|  | **Fully-adjusted model*** |  |
| --- | --- | --- |
| **Addictive behaviors** | ***P*** |  |
| **Tobacco use** |  |  |
| *Relapse of tobacco use among ex-smokers at baseline* |  |  |
| Physical exertion*occupational grade | 0.34 |  |
| Physical exertion* type of work | 0.23 |  |
| Physical exertion* type of job contract | 0.57 |  |
| *Changing status among current smokers at baseline* |  |  |
| Physical exertion*occupational grade | 0.07 |  |
| Physical exertion* type of work | 0.10 |  |
| Physical exertion* type of job contract | 0.66 |  |
| *Changing status among ever-smokers at baseline* |  |  |
| Physical exertion*occupational grade | 0.06 |  |
| Physical exertion* type of work | 0.42 |  |
| Physical exertion* type of job contract | 0.27 |  |
| *Number of cigarettes/day among current smokers at baseline* |  |  |
| Physical exertion*occupational grade | 0.06 |  |
| Physical exertion* type of work | 0.19 |  |
| Physical exertion* type of job contract | 0.53 |  |
|  |  |  |
| **Cannabis use** |  |  |
| *Relapse among ever-users at baseline* |  |  |
| Physical exertion*occupational grade | 0.08 |  |
| Physical exertion* type of work | 0.49 |  |
| Physical exertion* type of job contract | 0.75 |  |
|  |  |  |
| **Alcohol use** |  |  |
| *Number of glasses/week* |  |  |
| Physical exertion*occupational grade | 0.81 |  |
| Physical exertion* type of work | 0.20 |  |
| Physical exertion* type of job contract | 0.41 |  |
|  |  |  |
| **Diet rich in sugar and fat** |  |  |
| Physical exertion*occupational grade | 0.52 |  |
| Physical exertion* type of work | 0.81 |  |
| Physical exertion* type of job contract | 0.74 |  |
| *Adjusted for age (years, continuous), sex, occupational grade (low; medium; high), depressive symptoms at baseline (no; yes), educational level (levels, continuous), household income (€/month, continuous), baseline level of consumption, type of work time (part-time; full-time) and type of job contract (temporary; permanent). | | |
| Categories of current smokers were defined as: light smokers (<10 cigarettes/day), moderate smokers (10-18 cigarettes/day) and heavy smokers (>19 cigarettes/day). | | |
| Relapse was defined as: no (remained non-smokers at follow-up) and yes (became current smokers at follow-up). | | |
| Changing status among current smokers was defined as ex-smokers (stopped smoking at follow-up), current light smokers (remained current light smokers at follow-up), current moderate smokers (remained current moderate smokers at follow-up) and current heavy smokers (remained current heavy smokers at follow-up). | | |
