# Supplementary material for: Physical exertion at work and addictive behaviors: tobacco, cannabis, alcohol, sugar and fat consumption: longitudinal analyses in the CONSTANCES cohort
Source: Sci Rep. 2022 Jan 13;12:661. doi: 10.1038/s41598-021-04475-2 (PMC8758679; doi:10.1038/s41598-021-04475-2)
Supplement: Supplementary file 14 — Supplementary Table S13. [file 41598_2021_4475_MOESM14_ESM.docx]

**Supplementary Table S13.** Association between high physical exertion at work and addictive behaviors at one-year of follow-up according to occupational grade among employees in the CONSTANCES cohort study, 2012-2018 (odds ratios (ORs), and 95% confidence intervals, CI).

|  | **Low** | | | **Medium** | | | **High** |  |  |
| --- | --- | --- | --- | --- | --- | --- | --- | --- | --- |
|  |  | **Unadjusted model** | **Fully-adjusted model*** |  | **Unadjusted model** | **Fully-adjusted model*** |  | **Unadjusted model** | **Fully-adjusted model*** |
| **Addictive behaviors** | **N (%)** | **OR (95% CI)** | **OR (95% CI)** | **N (%)** | **OR (95% CI)** | **OR (95% CI)** | **N (%)** | **OR (95% CI)** | **OR (95% CI)** |
| **Tobacco use** |  |  |  |  |  |  |  |  |  |
| *Relapse of tobacco use among ex-smokers at baseline* |  |  |  |  |  |  |  |  |  |
| No | 8,878 (77.1) | 1.00 | 1.00 | 7,796 (83.0) | 1.00 | 1.00 | 8,544 (85.0) | 1.00 | 1.00 |
| Yes | 2,608 (22.9) | **1.21 (1.10-1.32)** | 1.06 (0.91-1.24) | 1,562 (17.0) | **1.19 (1.07-1.34)** | 1.13 (0.99-1.27) | 1,528 (15.0) | **1.20 (1.01-1.41)** | 1.27 (0.98-1.66) |
|  |  |  |  |  |  |  |  |  |  |
| *Changing status among current smokers at baseline* | 9,409 |  |  | 5,287 |  |  | 5,382 |  |  |
| Ex-smoker | 2,006 (21.3) | 1.00 | 1.00 | 1,686 (31.9) | 1.00 | 1.00 | 2,095 (38.9) | 1.00 | 1.00 |
| Current light smoker | 4,023 (42.7) | **1.39 (1.25-1.55)** | **1.21 (1.07-1.35)** | 2,233 (42.2) | **1.16 (1.02-1.32)** | **1.12 (1.00-1.28)** | 2150 (40.0) | **1.39 (1.14-1.70)** | **1.30 (1.06-1.59)** |
| Current moderate Smoker | 2,733 (29.0) | **1.89 (1.68-2.12)** | **1.43 (1.26-1.62)** | 1,128 (21.3) | **1.16 (1.00-1.36)** | **1.08 (1.00-1.26)** | 890 (16.5) | **1.73 (1.36-2.21)** | **1.48 (1.16-1.90)** |
| Current heavy smoker | 647 (7.0) | **2.43 (2.02-2.92)** | **1.62 (1.33-1.97)** | 240 (4.6) | **1.33 (1.01-1.75)** | **1.27 (1.01-1.68)** | 247 (4.6) | **1.99 (1.37-2.90)** | **1.58 (1.08-2.32)** |
| *P-trend* | **<0.0001** |  |  | **<0.0001** |  |  | **<0.0001** |  |  |
|  |  |  |  |  |  |  |  |  |  |
| *Changing status among ever-smokers at baseline* |  |  |  |  |  |  |  |  |  |
| Smoker at baseline and remained smoker at follow-up | 7,403 (35.6) | 1.00 | 1.00 | 3,600 (24.6) | 1.00 | 1.00 | 3,288 (21.5) | 1.00 | 1.00 |
| Smoker at baseline and stopped at follow-up | 2,006 (9.6) | **0.61 (0.56-0.68)** | **0.76 (0.69-0.85)** | 1,686 (11.5) | **0.85 (0.76-0.96)** | **0.89 (0.79-1.01)** | 2,095 (13.5) | **0.66 (0.55-0.79)** | **0.73 (0.61-0.88)** |
| Ex-smoker at baseline and stopped at follow-up | 8,788 (42.2) | **0.70 (0.66-0.74)** | **0.84 (0.79-0.91)** | 7,796 (53.2) | **0.77 (0.71-0.84)** | **0.89 (0.82-0.97)** | 8,544 (55.5) | **0.82 (0.72-0.92)** | **0.86 (0.75-0.98)** |
| Ex-smoker at baseline and started smoking at follow-up | 2,608 (12.5) | **0.84 (0.77-0.92)** | 0.89 (0.80-1.01) | 1,562 (10.7) | **0.93 (0.82-1.05)** | 1.00 (0.89-1.13) | 1,528 (9.8) | 0.98 (0.81-1.18) | 1.02 (0.82-1.23) |
| *P-trend* | **<0.0001** |  |  | **<0.0001** |  |  | **<0.0001** |  |  |
|  |  |  |  |  |  |  |  |  |  |
|  |  | ***ß* (95%CI)** | ***ß* (95%CI)** | 5,287 | ***ß* (95%CI)** | ***ß* (95%CI)** | 5,382 | ***ß* (95%CI)** | ***ß* (95%CI)** |
| *Number of cigarettes/day among current smokers at baseline* | 9,409 | -0.11 (-0.34;0.12) | **0.41 (0.20-0.63)** |  | -0.09 (-0.40;0.21) | **0.07 (0.20;0.34)** |  | **0.09 (-0.39;0.57)** | **0.44 (0.03-0.85)** |
|  |  |  |  |  |  |  |  |  |  |
| **Cannabis use** |  | **OR (95% CI)** | **OR (95% CI)** |  | **OR (95% CI)** | **OR (95% CI)** |  | **OR (95% CI)** | **OR (95% CI)** |
| *Relapse among ever-users at baseline* |  |  |  |  |  |  |  |  |  |
| No consumption in the past 12 months at follow-up | 10,532 (94.4) | 1.00 | 1.00 | 9,692 (95.2) | 1.00 | 1.00 | 12,107 (94.0) | 1.00 | 1.00 |
| In the past 12 months, <1/month | 477 (4.3) | 0.92 (0.76-1.10) | 0.88 (0.72-1.08) | 407 (4.0) | **0.98 (0.79-1.20)** | 0.94 (0.76-1.16) | 674 (5.2) | 1.06 (0.82-1.37) | 1.07 (0.82-1.39) |
| In the past 12 months, ≥1/month | 152 (1.4) | **1.89 (1.37-2.62)** | 1.62 (0.99-2.29) | 85 (0.8) | **0.93 (0.60-1.42)** | 0.88 (0.57-1.37) | 102 (0.8) | 1.43 (0.79-2.57) | 1.28 (0.71-2.32) |
|  |  |  |  |  |  |  |  |  |  |
|  |  | **OR (95% CI)** | **OR (95% CI)** |  | **OR (95% CI)** | **OR (95% CI)** |  | **OR (95% CI)** | **OR (95% CI)** |
| **Alcohol use** |  |  |  |  |  |  | 25233 |  |  |
| Low risk | 17,805 (62.9) | 1.00 | 1.00 | 14,136 (64.9) | 1.00 | 1.00 | 17,859 (70.8) | 1.00 | 1.00 |
| No use | 6,555 (23.2) | **0.84 (0.79-0.89)** | 0.95 (0.89-1.01) | 4,947 (22.4) | **1.15 (1.07-1.22)** | 1.06 (0.99-1.14) | 4,260 (16.9) | **1.19 (1.07-1.33)** | 1.06 (0.95-1.19) |
| At risk | 3,929 (13.9) | **0.90 (0.84**  **-0.97)** | 1.01 (0.93-1.09) | 2,809 (12.7) | **1.12 (1.03-1.22)** | 1.09 (0.99-1.20) | 3,114 (12.3) | **1.15 (1.02-1.30)** | 1.01 (0.89-1.16) |
|  |  |  |  |  |  |  |  |  |  |
|  |  | ***ß* (95%CI)** | ***ß* (95%CI)** |  | ***ß* (95%CI)** | ***ß* (95%CI)** |  | ***ß* (95%CI)** | ***ß* (95%CI)** |
| *Number of glasses/week* |  | -0.03 (-0.27;0.20) | 0.14 (-0.08;0.37) | 22,062 | 0.06 (-0.19;0.31) | 0.12 (-0.12;0.35) | 25,063 | 0.06 (-0.32;0.44) | 0.07 (-0.28;0.42) |
|  | 28,289 |  |  |  |  |  |  |  |  |
| **Diet rich in sugar and fat** |  | **OR (95% CI)** | **OR (95% CI)** |  | **OR (95% CI)** | **OR (95% CI)** |  | **OR (95% CI)** | **OR (95% CI)** |
| First quartile | 7108 (25.2) | 1.00 | 1.00 | 5,524 (25.0) | 1.00 | 1.00 | 6,072 (24.2) | 1.00 | 1.00 |
| Second quartile | 7,112 (25.1) | 1.05 (0.99-1.12) | 1.01 (0.94-1.09) | 5,553 (25.1) | 1.10 (1.02-1.19) | 1.11 (1.03 (1.21) | 6,338 (25.3) | 0.93 (0.83-1.04) | 1.09 (0.89-1.13) |
| Third quartile | 7,149 (25.3) | **1.15 (1.07-1.22)** | 1.06 (0.99-1.14) | 5,457 (24.9) | 1.07 (0.99-1.15) | 1.06 (0.98-1.15) | 6,248 (24.9) | 0.96 (0.86-1.08) | 1.09 (0.97-1.23) |
| Fourth quartile | 6,920 (24.4) | **1.31 (1.22-1.40)** | **1.15 (1.07-1.24)** | 5,528 (25.0) | **1.12 (1.04-1.21)** | **1.10 (1.01-1.19)** | 6,405 (25.6) | 0.95 (0.87-1.10) | **1.18 (1.05-1.33)** |
| *P-trend* | **<0.0001** |  |  | **<0.0001** |  |  | **<0.0001** |  |  |

| *Adjusted for age (years, continuous), depressive symptoms at baseline (no; yes), educational level (levels, continuous), household income (€/month, continuous) and baseline level of consumption. |
| --- |
| Categories of current smokers were defined as: light smokers (<10 cigarettes/day), moderate smokers (10-18 cigarettes/day) and heavy smokers (>19 cigarettes/day). |
| Relapse was defined as: no (remained non-smokers at follow-up) and yes (became current smokers at follow-up). |
| Changing status among current smokers was defined as ex-smokers (stopped smoking at follow-up), current light smokers (remained current light smokers at follow-up), current moderate smokers (remained current moderate smokers at follow-up) and current heavy smokers (remained current heavy smokers at follow-up).  Alcohol use was defined as: low risk (1-27 drinks/week in men and 1-13 i\n women); no use and at risk (≥28 drinks/week in men and ≥14 in women). |
