# Supplementary material for: Physical exertion at work and addictive behaviors: tobacco, cannabis, alcohol, sugar and fat consumption: longitudinal analyses in the CONSTANCES cohort
Source: Sci Rep. 2022 Jan 13;12:661. doi: 10.1038/s41598-021-04475-2 (PMC8758679; doi:10.1038/s41598-021-04475-2)
Supplement: Supplementary file 15 — Supplementary Table S14. [file 41598_2021_4475_MOESM15_ESM.docx]

**Supplementary Table S14.** Association between high physical exertion at work and addictive behaviors at one-year of follow-up according to the type of job contract among employees in the CONSTANCES cohort study, 2012-2018 (odds ratios (ORs), and 95% confidence intervals, CI).

|  | **Temporary contract** | | |  | **Permanent contract** | | |
| --- | --- | --- | --- | --- | --- | --- | --- |
|  |  | **Unadjusted model** | **Fully-adjusted model*** |  |  | **Unadjusted model** | **Fully-adjusted model*** |
| **Addictive behaviors** | **N (%)** | **OR (95% CI)** | **OR (95% CI)** |  | **N (%)** | **OR (95% CI)** | **OR (95% CI)** |
| **Tobacco use** |  |  |  |  |  |  |  |
| *Relapse of tobacco use among ex-smokers at baseline* | 9,900 |  |  |  | 21,016 |  |  |
| No | 8,004 (80.9) | 1.00 | 1.00 |  | 17,214 (81.9) | 1.00 | 1.00 |
| Yes | 1,896 (19.1) | **1.31 (1.18-1.46)** | 1.07 (0.87-1.31) |  | 3,802 (18.1) | **1.39 (1.30-1.50)** | 1.15 (0.99-1.29) |
|  |  |  |  |  |  |  |  |
| *Changing status among current smokers at baseline* | 7,071 |  |  |  | 13,007 |  |  |
| Ex-smoker | 2,020 (28.5) | 1.00 | 1.00 |  | 3,767 (29.0) | 1.00 | 1.00 |
| Current light smoker | 3,004 (42.5) | **1.48 (1.32-1.67)** | **1.18 (1.04-1.35)** |  | 5,402 (41.5) | **1.58 (1.44-1.72)** | **1.23 (1.12-1.36)** |
| Current moderate Smoker | 1,675 (23.7) | **2.07 (1.81-2.36)** | **1.32 (1.14-1.54)** |  | 3,076 (23.6) | **2.18 (1.97-2.41)** | **1.36 (1.21-1.52)** |
| Current heavy smoker | 372 (5.3) | **2.52 (2.02-3.15)** | **1.61 (1.26-2.06)** |  | 762 (5.9) | **2.45 (2.08-2.87)** | **1.50 (1.25-1.80)** |
| *P-trend* | **<0.0001** |  |  |  | **<0.0001** |  |  |
|  |  |  |  |  |  |  |  |
| *Changing status among ever-smokers at baseline* | 16,971 |  |  |  | 34,023 |  |  |
| Smoker at baseline and remained smoker at follow-up | 5,051 (29.8) | 1.00 | 1.00 |  | 9,240 (27.2) | 1.00 | 1.00 |
| Smoker at baseline and stopped at follow-up | 2,020 (11.9) | **0.58 (0.52-0.65)** | **0.80 (0.71-0.90)** |  | 3,767 (11.0) | **0.55 (0.50-0.59)** | **0.77 (0.70-0.84)** |
| Ex-smoker at baseline and stopped at follow-up | 8,004 (47.1) | **0.59 (0.55-0.63)** | **0.84 (0.77-0.91)** |  | 17,214 (50.6) | **0.62 (0.58-0.65)** | **0.86 (0.81-0.91)** |
| Ex-smoker at baseline and started smoking at follow-up | 1,896 (11.2) | **0.77 (0.69-0.86)** | 0.89 (0.79-1.01) |  | 3,802 (11.2) | **0.86 (0.79-0.93)** | 0.96 (0.88-1.05) |
| *P-trend* | **<0.0001** |  |  |  | **<0.0001** |  |  |
|  |  |  |  |  |  |  |  |
|  |  | ***ß* (95%CI)** | ***ß* (95%CI)** |  | 13,007 | ***ß* (95%CI)** | ***ß* (95%CI)** |
| *Number of cigarettes/day among current smokers at baseline* | 7,071 | 0.04 (-0.22;0.30) | **0.36 (0.11-0.62)** |  |  | -0.07 (-0.27;0.13) | **0.34 (0.15-0.54)** |
|  |  |  |  |  |  |  |  |
| **Cannabis use** |  | **OR (95% CI)** | **OR (95% CI)** |  |  | **OR (95% CI)** | **OR (95% CI)** |
| *Relapse among ever-users at baseline* | 12,566 |  |  |  | 21,662 |  |  |
| No consumption in the past 12 months at follow-up | 11,837 (94.2) | 1.00 | 1.00 |  | 20,494 (94.6) | 1.00 | 1.00 |
| In the past 12 months, <1/month | 588 (4.7) | 0.97 (0.81-1.16) | 1.00 (0.81-1.22) |  | 970 (4.5) | **0.85 (0.74-0.99)** | 0.89 (0.76-1.05) |
| In the past 12 months, ≥1/month | 141 (1.1) | **1.57 (1.13-2.20)** | 1.25 (0.86-1.80) |  | 198 (0.9) | **1.72 (1.31-2.27)** | 1.35 (0.99-1.84) |
|  |  |  |  |  |  |  |  |
|  |  | **OR (95% CI)** | **OR (95% CI)** |  |  | **OR (95% CI)** | **OR (95% CI)** |
| **Alcohol use** |  |  |  |  |  |  |  |
| Low risk | 15,675 (64.7) | 1.00 | 1.00 |  | 34,125 (66.7) | 1.00 | 1.00 |
| No use | 5,318 (22.0) | 1.05 (0.98-1.12) | 0.97 (0.90-1.04) |  | 10,444 (20.4) | **1.17 (1.12-1.23)** | 1.04 (0.99-1.10) |
| At risk | 3,229 (13.3) | 1.07 (0.99-1.16) | 1.09 (0.99-1.19) |  | 6,623 (12.9) | **1.10 (1.04-1.16)** | 1.02 (0.95-1.09) |
|  |  |  |  |  |  |  |  |
|  |  | ***ß* (95%CI)** | ***ß* (95%CI)** |  |  | ***ß* (95%CI)** | ***ß* (95%CI)** |
| *Number of glasses/week* | 24,222 | 0.18 (-0.08;0.43) | 0.24 (-0.01;0.49) |  | 51,192 | -0.05 (-0.23;0.12) | 0.06 (-0.12;0.24) |
|  |  |  |  |  |  |  |  |
| **Diet rich in sugar and fat** |  | **OR (95% CI)** | **OR (95% CI)** |  |  | **OR (95% CI)** | **OR (95% CI)** |
| First quartile | 5,907 (24.4) | 1.00 | 1.00 |  | 12,797 (25.0) | 1.00 | 1.00 |
| Second quartile | 6,123 (25.3) | 1.06 (0.98-1.14) | 1.06 (0.97-1.15) |  | 12,880 (25.2) | 1.01 (0.96-1.07) | 1.03 (0.97-1.09) |
| Third quartile | 6,057 (25.0) | **1.13 (1.04-1.22)** | 1.08 (0.99-1.18) |  | 12,797 (25.0) | 1.05 (0.99-1.10) | 1.05 (0.99-1.11) |
| Fourth quartile | 6,135 (25.0) | **1.17 (1.08-1.26)** | **1.12 (1.02-1.22)** |  | 12,718 (24.8) | **1.10 (1.05-1.16)** | **1.13 (1.06-1.20)** |
| *P-trend* | **<0.0001** |  |  |  | **<0.0001** |  |  |

| *Adjusted for age (years, continuous), occupational grade (low; medium; high), depressive symptoms at baseline (no; yes), educational level (levels, continuous), household income (€/month, continuous) and baseline level of consumption. |
| --- |
| Categories of current smokers were defined as: light smokers (<10 cigarettes/day), moderate smokers (10-18 cigarettes/day) and heavy smokers (>19 cigarettes/day). |
| Relapse was defined as: no (remained non-smokers at follow-up) and yes (became current smokers at follow-up). |
| Changing status among current smokers was defined as ex-smokers (stopped smoking at follow-up), current light smokers (remained current light smokers at follow-up), current moderate smokers (remained current moderate smokers at follow-up) and current heavy smokers (remained current heavy smokers at follow-up).  Alcohol use was defined as: low risk (1-27 drinks/week in men and 1-13 i\n women); no use and at risk (≥28 drinks/week in men and ≥14 in women). |
