# Supplementary material for: Physical exertion at work and addictive behaviors: tobacco, cannabis, alcohol, sugar and fat consumption: longitudinal analyses in the CONSTANCES cohort
Source: Sci Rep. 2022 Jan 13;12:661. doi: 10.1038/s41598-021-04475-2 (PMC8758679; doi:10.1038/s41598-021-04475-2)
Supplement: Supplementary file 16 — Supplementary Table S15. [file 41598_2021_4475_MOESM16_ESM.docx]

**Supplementary Table S15.** Association between high physical exertion at work and addictive behaviors at one-year of follow-up according to the type of work among employees in the CONSTANCES cohort study, 2012-2018 (odds ratios (ORs), and 95% confidence intervals, CI).

|  | **Part-time** | | |  | **Full-time** | | |
| --- | --- | --- | --- | --- | --- | --- | --- |
|  |  | **Unadjusted model** | **Fully-adjusted model*** |  |  | **Unadjusted model** | **Fully-adjusted model*** |
| **Addictive behaviors** | **N (%)** | **OR (95% CI)** | **OR (95% CI)** |  | **N (%)** | **OR (95% CI)** | **OR (95% CI)** |
| **Tobacco use** |  |  |  |  |  |  |  |
| *Relapse of tobacco use among ex-smokers at baseline* | 4,910 |  |  |  | 26,006 |  |  |
| No | 3,990 (81.3) | 1.00 | 1.00 |  | 21,228 (81.6) | 1.00 | 1.00 |
| Yes | 920 (18.7) | **1.41 (1.22-1.64)** | 1.14 (0.95-1.36) |  | 4,778 (18.4) | **1.36 (1.27-1.45)** | 1.12 (0.98-1.24) |
|  |  |  |  |  |  |  |  |
| *Changing status among current smokers at baseline* | 3,160 |  |  |  | 16,918 |  |  |
| Ex-smoker | 871 (27.5) | 1.00 | 1.00 |  | 4,916 (29.0) | 1.00 | 1.00 |
| Current light smoker | 1,381 (43.7) | **1.41 (1.18-1.68)** | 1.18 (0.98-1.43) |  | 7,025 (41.5) | **1.57 (1.45-1.69)** | 1.22 (0.99-1.33) |
| Current moderate Smoker | 751 (23.8) | **1.92 (1.57-2.35)** | **1.40 (1.13-1.73)** |  | 4,000 (23.6) | **2.18 (2.00-2.38)** | **1.33 (1.20-1.47)** |
| Current heavy smoker | 157 (5.0) | **2.79 (1.97-3.95)** | **2.11 (1.46-3.06)** |  | 977 (5.8) | **2.43 (2.11-2.80)** | **1.44 (1.23-1.69)** |
| *P-trend* |  |  |  |  |  |  |  |
|  |  |  |  |  |  |  |  |
| *Changing status among ever-smokers at baseline* | 8,070 |  |  |  | 42,924 |  |  |
| Smoker at baseline and remained smoker at follow-up | 2,290 (28.4) | 1.00 | 1.00 |  | 12,002 (28.0) | 1.00 | 1.00 |
| Smoker at baseline and stopped at follow-up | 871 (10.8) | **0.61 (0.52-0.72)** | **0.79 (0.67-0.94)** |  | 4,916 (11.4) | **0.55 (0.51-0.59)** | **0.78 (0.72-0.84)** |
| Ex-smoker at baseline and stopped at follow-up | 3,990 (49.4) | **0.61 (0.55-0.68)** | 0.89 (0.79-1.00) |  | 21,228 (49.5) | **0.60 (0.58-0.63)** | 0.84 (0.80-1.01) |
| Ex-smoker at baseline and started smoking at follow-up | 920 (11.4) | **0.86 (0.74-1.01)** | 1.00 (0.85-1.18) |  | 4,778 (11.1) | **0.82 (0.76-0.88)** | 0.92 (0.85-1.00) |
| *P-trend* | **<0.0001** |  |  |  | **<0.0001** |  |  |
|  |  |  |  |  |  |  |  |
|  |  | ***ß* (95%CI)** | ***ß* (95%CI)** |  |  | ***ß* (95%CI)** | ***ß* (95%CI)** |
| *Number of cigarettes/day among current smokers at baseline* | 3,160 | 0.26 (-0.13;0.65) | 0.68 (0.33-1.04) |  | 16,918 | -0.09 (-0.26;0.09) | 0.27 (0.10-0.45) |
|  |  |  |  |  |  |  |  |
| **Cannabis use** |  | **OR (95% CI)** | **OR (95% CI)** |  |  | **OR (95% CI)** | **OR (95% CI)** |
| *Relapse among ever-users at baseline* | 4,719 |  |  |  | 29,509 |  |  |
| No consumption in the past 12 months at follow-up | 4,453 (94.4) | 1.00 | 1.00 |  | 27,878 (94.5) | 1.00 | 1.00 |
| In the past 12 months, <1/month | 211 (4.5) | 1.11 (0.83-1.49) | 1.08 (0.79-1.48) |  | 1,347 (4.6) | **0.86 (0.76-0.97)** | 0.90 (0.78-1.04) |
| In the past 12 months, ≥1/month | 55 (1.1) | 1.45 (0.87-2.42) | 0.96 (0.56-1.64) |  | 284 (0.9) | **1.70 (1.35-2.15)** | 1.21 (0.92-1.59) |
|  |  |  |  |  |  |  |  |
|  |  | **OR (95% CI)** | **OR (95% CI)** |  |  | **OR (95% CI)** | **OR (95% CI)** |
| **Alcohol use** |  |  |  |  |  |  |  |
| Low risk | 7,237 (58.9) | 1.00 | 1.00 |  | 42,563 (67.4) | 1.00 | 1.00 |
| No use | 3,142 (25.5) | 0.99 (0.91-1.08) | 0.89 (0.81-1.03) |  | 12,620 (20.0) | **1.15 (1.10-1.20)** | 1.05 (0.98-1.10) |
| At risk | 1,916 (15.6) | 0.97 (0.88-1.08) | 0.92 (0.81-1.03) |  | 7,936 (12.6) | **1.10 (1.05-1.16)** | 1.07 (0.99-1.14) |
|  |  |  |  |  |  |  |  |
|  |  | ***ß* (95%CI)** | ***ß* (95%CI)** |  |  | ***ß* (95%CI)** | ***ß* (95%CI)** |
| *Number of glasses/week* | 12,295 | -0.01 (-0.34;0.31) | 0.06 (-0.26;0.38) |  | 63,119 | 0.02 (-0.14;0.19) | 0.13 (-0.03;0.29) |
|  |  |  |  |  |  |  |  |
| **Diet rich in sugar and fat** |  | **OR (95% CI)** | **OR (95% CI)** |  |  | **OR (95% CI)** | **OR (95% CI)** |
| First quartile | 3,135 (25.5) | 1.00 | 1.00 |  | 15,569 (24.7) | 1.00 | 1.00 |
| Second quartile | 2,951 (24.0) | 1.04 (0.94-1.15) | 1.07 (0.96-1.19) |  | 16,052 (25.4) | 1.03 (0.98-1.08) | 1.03 (0.96-1.09) |
| Third quartile | 3,074 (25.0) | 1.03 (0.93-1.14) | 1.06 (0.95-1.18) |  | 15,780 (25.0) | **1.09 (1.04-1.14)** | 1.06 (0.99-1.12) |
| Fourth quartile | 3,135 (25.5) | **1.12 (1.01-1.24)** | **1.17 (1.05-1.32)** |  | 15,718 (24.9) | **1.13 (1.08-1.19)** | **1.11 (1.06-1.18)** |
| *P-trend* | **<0.0001** |  |  |  | **<0.0001** |  |  |

| *Adjusted for age (years, continuous), occupational grade (low; medium; high), depressive symptoms at baseline (no; yes), educational level (levels, continuous), household income (€/month, continuous) and baseline level of consumption. |
| --- |
| Categories of current smokers were defined as: light smokers (<10 cigarettes/day), moderate smokers (10-18 cigarettes/day) and heavy smokers (>19 cigarettes/day). |
| Relapse was defined as: no (remained non-smokers at follow-up) and yes (became current smokers at follow-up). |
| Changing status among current smokers was defined as ex-smokers (stopped smoking at follow-up), current light smokers (remained current light smokers at follow-up), current moderate smokers (remained current moderate smokers at follow-up) and current heavy smokers (remained current heavy smokers at follow-up).  Alcohol use was defined as: low risk (1-27 drinks/week in men and 1-13 i\n women); no use and at risk (≥28 drinks/week in men and ≥14 in women). |
