# Supplementary material for: Physical exertion at work and addictive behaviors: tobacco, cannabis, alcohol, sugar and fat consumption: longitudinal analyses in the CONSTANCES cohort
Source: Sci Rep. 2022 Jan 13;12:661. doi: 10.1038/s41598-021-04475-2 (PMC8758679; doi:10.1038/s41598-021-04475-2)
Supplement: Supplementary file 17 — Supplementary Table S16. [file 41598_2021_4475_MOESM17_ESM.docx]

**Supplementary Table S16.** Association between high physical exertion and addictive behaviors (odds ratios (ORs), 95% confidence intervals, CI).

|  |  | **Unadjusted model** | **Fully-adjusted model*** |  |
| --- | --- | --- | --- | --- |
| **Addictive behaviors** | **N (%)** | **OR (95% CI)** | **OR (95% CI)** |  |
| **Tobacco use** |  |  |  |  |
| *Relapse of tobacco use among ex-smokers at baseline* | 30,916 |  |  |  |
| No | 25,218 (81.6) | 1.00 | 1.00 |  |
| Yes | 5,698 (18.4) | **1.37 (1.29-1.45)** | **1.12 (1.01-1.23)** |  |
|  |  |  |  |  |
| *Changing status among current smokers at baseline* | 20,078 |  |  |  |
| Ex-smoker | 5,787 (28.8) | 1.00 | 1.00 |  |
| Current light smoker | 8,406 (41.9) | **1.54 (1.43-1.66)** | **1.19 (1.10-1.29)** |  |
| Current moderate Smoker | 4,751 (23.7) | **2.14 (1.97-2.32)** | **1.32 (1.21-1.44)** |  |
| Current heavy smoker | 1,134 (5.6) | **2.47 (2.17-2.81)** | **1.49 (1.29-1.72)** |  |
| *P-trend* | **<0.0001** |  |  |  |
|  |  |  |  |  |
| *Changing status among ever-smokers at baseline* | 50,994 |  |  |  |
| Smoker at baseline and remained smoker at follow-up | 14,291 (28.0) | 1.00 | 1.00 |  |
| Smoker at baseline and stopped at follow-up | 5,787 (11.3) | **0.56 (0.52-0.60)** | **0.79 (0.74-0.85)** |  |
| Ex-smoker at baseline and stopped at follow-up | 25,218 (49.5) | **0.60 (0.58-0.63)** | **0.88 (0.84-0.93)** |  |
| Ex-smoker at baseline and started smoking at follow-up | 5,698 (11.2) | **0.83 (0.78-0.88)** | 0.96 (0.90-1.03) |  |
| *P-trend* | **<0.0001** |  |  |  |
|  |  |  |  |  |
|  |  | ***ß* (95%CI)** | ***ß* (95%CI)** |  |
| *Number of cigarettes/day among current smokers at baseline* | 20,078 | -0.02 (-0.18;0.14) | **0.32 (0.17;0.48)** |  |
|  |  |  |  |  |
| **Cannabis use** |  | **OR (95% CI)** | **OR (95% CI)** |  |
| *Relapse among ever-users at baseline* | 34,228 |  |  |  |
| No consumption in the past 12 months at follow-up | 32,331 (94.5) | 1.00 | 1.00 |  |
| In the past 12 months, <1/month | 1,558 (4.5) | 0.90 (0.80-1.00) | 0.89 (0.79-1.01) |  |
| In the past 12 months, ≥1/month | 339 (1.0) | **1.67 (1.35-2.06)** | **1.25 (1.00-1.58)** |  |
|  |  |  |  |  |
| **Alcohol use** |  |  |  |  |
| Low risk | 49,800 (66.0) | 1.00 | 1.00 |  |
| No use | 15,762 (20.9) | **1.13 (1.09-1.17)** | 1.02 (0.97-1.03) |  |
| At risk | 9,852 (13.1) | **1.09 (1.04-1.14)** | 1.03 (0.98-1.09) |  |
|  |  |  |  |  |
|  |  | ***ß* (95%CI)** | ***ß* (95%CI)** |  |
| *Number of glasses/week* | 75,414 | **0.02 (0.13;0.16)** | 0.11 (-0.04;025) |  |
|  |  |  |  |  |
| **Diet rich in sugar and fat** |  | **OR (95% CI)** | **OR (95% CI)** |  |
| First quartile | 18,704 (24.8) | 1.00 | 1.00 |  |
| Second quartile | 19,003 (25.2) | 1.03 (0.98-1.07) | 1.05 (0.99-1.10) |  |
| Third quartile | 18,854 (25.0) | **1.07 (1.03-1.12)** | **1.07 (1.02-1.13)** |  |
| Fourth quartile | 18,853 (25.0) | **1.12 (1.08-1.17)** | **1.15 (1.09-1.20)** |  |
| *P-trend* | **<0.0001** |  |  |  |
| *Adjusted for age (years, continuous), sex, depressive symptoms at baseline (no; yes), socioeconomic status (status, continuous) and baseline level of consumption. | | | | |
| Categories of current smokers were defined as: light smokers (<10 cigarettes/day), moderate smokers (10-18 cigarettes/day) and heavy smokers (>19 cigarettes/day). | | | | |
| Relapse was defined as: no (remained non-smokers at follow-up) and yes (became current smokers at follow-up). | | | | |
| Changing status among current smokers was defined as ex-smokers (stopped smoking at follow-up), current light smokers (remained current light smokers at follow-up), current moderate smokers (remained current moderate smokers at follow-up) and current heavy smokers (remained current heavy smokers at follow-up).  Alcohol use was defined as: low risk (1-27 drinks/week in men and 1-13 in women); no use and at risk (≥28 drinks/week in men and ≥14 in women). | | | | |
